# Odorant Metabolism Catalyzed by Olfactory Mucosal Enzymes Influences Peripheral Olfactory Responses in Rats

**DOI:** 10.1371/journal.pone.0059547

**Published:** 2013-03-26

**Authors:** Nicolas Thiebaud, Stéphanie Veloso Da Silva, Ingrid Jakob, Gilles Sicard, Joëlle Chevalier, Franck Ménétrier, Olivier Berdeaux, Yves Artur, Jean-Marie Heydel, Anne-Marie Le Bon

**Affiliations:** 1 CNRS, UMR6265, Centre des Sciences du Goût et de l’Alimentation, Dijon, France; 2 INRA, UMR1324, Centre des Sciences du Goût et de l’Alimentation, Dijon, France; 3 Université de Bourgogne, Centre des Sciences du Goût et de l’Alimentation, Dijon, France; 4 Université Aix-Marseille 2, UMR7259, Neurobiologie des Interactions Cellulaires et Neurophysiopathologie, Marseille, France; Duke University, United States of America

## Abstract

A large set of xenobiotic-metabolizing enzymes (XMEs), such as the cytochrome P450 monooxygenases (CYPs), esterases and transferases, are highly expressed in mammalian olfactory mucosa (OM). These enzymes are known to catalyze the biotransformation of exogenous compounds to facilitate elimination. However, the functions of these enzymes in the olfactory epithelium are not clearly understood. In addition to protecting against inhaled toxic compounds, these enzymes could also metabolize odorant molecules, and thus modify their stimulating properties or inactivate them. In the present study, we investigated the *in vitro* biotransformation of odorant molecules in the rat OM and assessed the impact of this metabolism on peripheral olfactory responses. Rat OM was found to efficiently metabolize quinoline, coumarin and isoamyl acetate. Quinoline and coumarin are metabolized by CYPs whereas isoamyl acetate is hydrolyzed by carboxylesterases. Electro-olfactogram (EOG) recordings revealed that the hydroxylated metabolites derived from these odorants elicited lower olfactory response amplitudes than the parent molecules. We also observed that glucurono-conjugated derivatives induced no olfactory signal. Furthermore, we demonstrated that the local application of a CYP inhibitor on rat olfactory epithelium increased EOG responses elicited by quinoline and coumarin. Similarly, the application of a carboxylesterase inhibitor increased the EOG response elicited by isoamyl acetate. This increase in EOG amplitude provoked by XME inhibitors is likely due to enhanced olfactory sensory neuron activation in response to odorant accumulation. Taken together, these findings strongly suggest that biotransformation of odorant molecules by enzymes localized to the olfactory mucosa may change the odorant’s stimulating properties and may facilitate the clearance of odorants to avoid receptor saturation.

## Introduction

In mammals, the process of olfaction begins in the olfactory epithelium with the binding of odorant molecules to membrane receptors expressed by olfactory sensory neurons (OSNs). This interaction triggers intracellular reaction cascades that transduce the chemical signal into electrical activity, which is then conveyed to the brain for further processing. There is growing evidence that the activation of olfactory receptors (ORs) can be influenced by biochemical events that occur in the vicinity of the OSNs. These perireceptor events may regulate the transport, residence time and clearance of odorants in the receptor environment [1], [2].

A number of proteins in the mucus covering the olfactory epithelium can catalyze these processes. Among them, odorant binding proteins (OBPs) may play an important role in the solubilization and transport of odorant molecules in the mucus [3]. Enzymes secreted in the mucus have been shown to biotransform such odorants as aldehydes and esters [4], [5]. Furthermore, numerous xenobiotic-metabolizing enzymes (XMEs) are highly expressed in mammalian olfactory mucosa (OM) [6], [7]. XMEs catalyze the biotransformation of a wide range of foreign molecules, called xenobiotics, and of many endogenous compounds. These enzymes often act sequentially. First, phase I enzymes (e.g., cytochrome P450 monooxygenases (CYPs), carboxylesterases, etc.) functionalize xenobiotics by forming polar metabolites. Then, phase II enzymes such as UDP-glucuronosyl transferases (UGTs) or glutathione-S-transferases conjugate metabolites with a polar moiety (e.g., UDP-glucuronic acid, glutathione, etc.) to increase compound hydrophilicity. The last step (phase III) involves transporters that facilitate the excretion of conjugated metabolites from the cell [8], [9].

The functions of XMEs found in the olfactory epithelium, however, are still not clearly understood. These enzymes most likely play a primary role in protecting the olfactory epithelium against inhaled chemicals [10]. They may also protect the brain because the olfactory nerve can carry viruses, bacteria and chemicals into the brain [11]–[13]. Furthermore, XMEs may play an active role in modulating olfactory input through metabolizing odorant molecules [14], [15]. This process could modify the olfactory-stimulating properties of the odorants.

Although this is a standing hypothesis, no study has clearly demonstrated that olfactory XMEs influence olfactory signals in mammals. In contrast, in insects, a growing set of data suggests that several enzymes such as esterases or aldehyde oxidases found in the sensilla lymph of antennae have the ability to metabolize odorant molecules and pheromones [4], [16], [17]. Interestingly, recent functional studies have shown that intracellular CYPs and carboxylesterases from scarab beetle and moth antennae can also catalyze the biotransformation of volatile compounds [18], [19]. Inhibition of CYPs by a specific inhibitor induces anosmia in the pheromone-detecting OSNs, demonstrating that CYPs are involved in pheromone metabolism [18]. Given the numerous similarities between insect and mammalian olfactory systems [20], these findings strongly support the possibility that olfactory XMEs may also modulate olfactory signals in mammals.

Thus, we designed the present study to assess the impact of phase I XMEs on olfactory epithelial responses to odorant stimulations in the rat. We studied the *in vitro* biotransformation of three odorant molecules (quinoline, coumarin and isoamyl acetate) to evaluate the metabolic capacity of the OM and to identify the products formed. Subsequently, we performed electroolfactogram (EOG) recordings to compare the intensity of the olfactory responses elicited by these odorant molecules and those elicited by their metabolites. Lastly, we examined the effects of *in situ* treatment with 1-aminobenzotriazole (ABT) and bis-*p*-nitro-phenylphosphate (BNPP), specific inhibitors of CYPs and carboxylesterases, respectively, on peripheral olfactory responses to these odorants. Our results demonstrated that XMEs localized to mammalian OM can efficiently biotransform odorants and modulate peripheral olfactory responses.

## Results

### *In vitro* Metabolism of Odorant Molecules

#### Metabolism of quinolone

**Figure 1:**
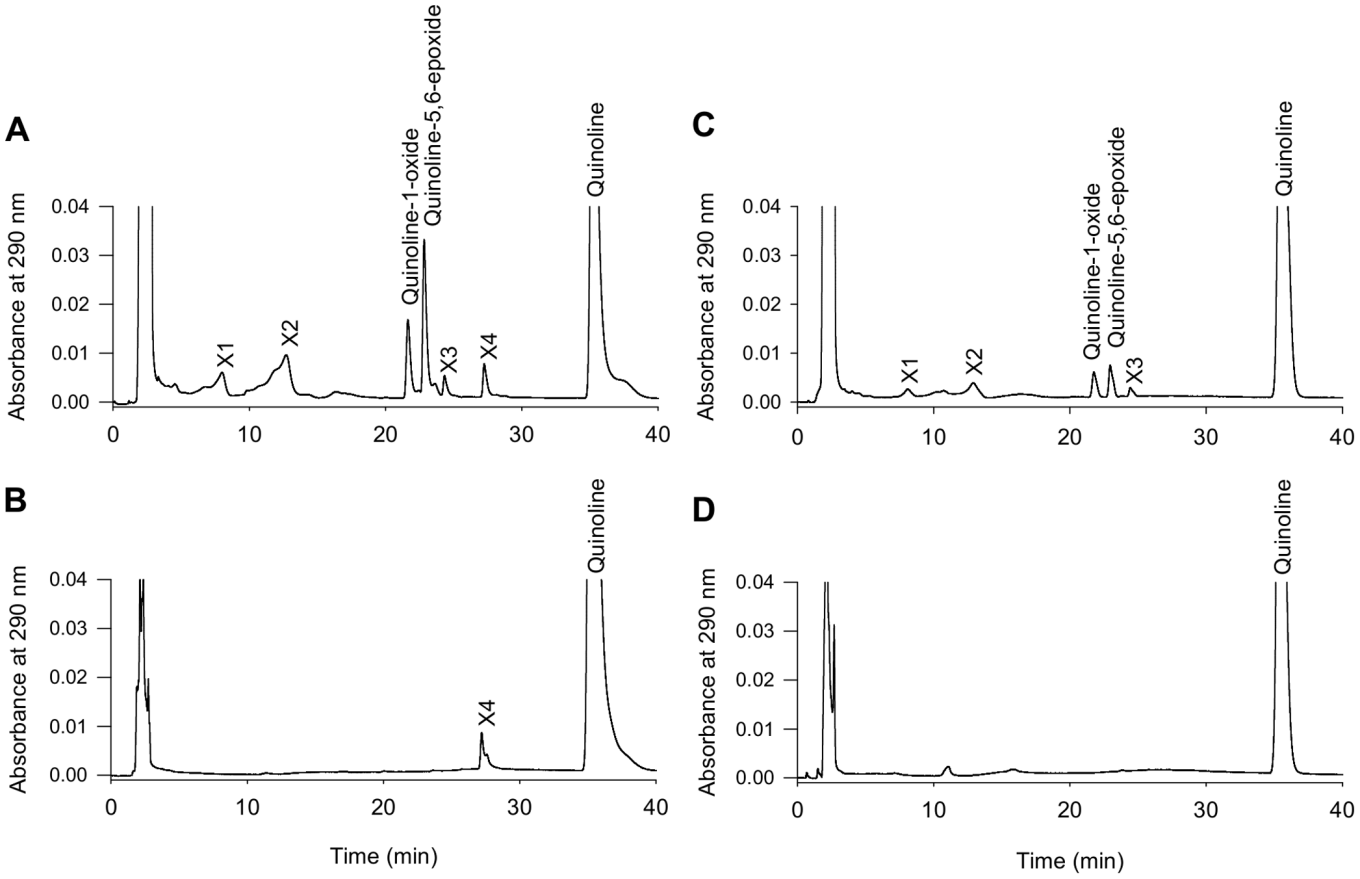
Representative HPLC profiles of quinoline metabolites formed *in vitro* by rat hepatic and olfactory microsomes. Four experimental conditions are shown: (A) reaction mixture containing olfactory microsomes and NADPH; (B) reaction mixture containing olfactory microsomes but no NADPH; (C) reaction mixture containing hepatic microsomes and NADPH; (D) reaction mixture containing hepatic microsomes but no NADPH.

**Figure 2:**
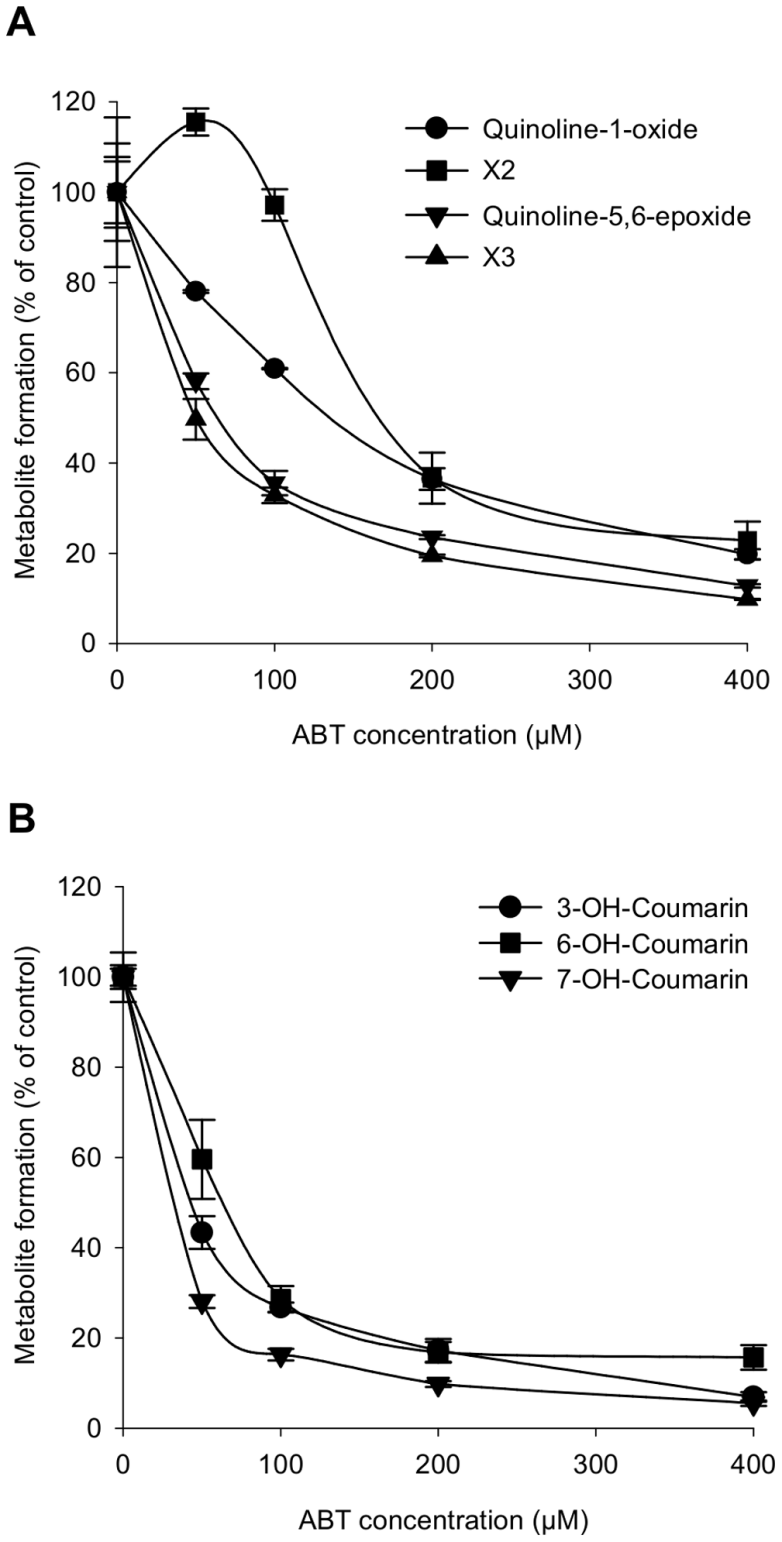
Inhibition of quinoline and coumarin microsomal metabolism by the CYP inhibitor ABT. (A) Effect of ABT on quinoline metabolism; (B) effect of ABT on coumarin metabolism. Values represent mean of 3 replicates ± S.E.M.

When quinoline was incubated with olfactory microsomes and NADPH, several metabolites were formed (Fig. 1A). Mass spectrometry analysis indicated that these compounds are oxygenated metabolites (mono-hydroxylated and diol derivatives) (Fig. S1). Quinoline-1-oxide and quinoline-5,6-epoxide appeared to be the major metabolites. Quinoline-1-oxide was identified by comparing its mass spectra with that of the standard compound (Fig. S2). Because the standard of quinoline-5,6-epoxide was not commercially available, this compound was identified by comparing its UV spectra to previously published spectra [21] and by taking into account its molecular mass given by mass spectrometry analysis. Mass spectrometry analysis also indicated that the peak X2 would correspond to diols (Fig. S3). With the exception of X4, no metabolite was formed when the incubation medium lacked NADPH (Fig. 1B). Hepatic microsomes produced a similar profile of metabolites, but the resulting biotransformation intensity was lower than that resulting from olfactory microsome incubation (Fig. 1C). No metabolites were detected when hepatic microsomes were incubated without NADPH (Fig. 1D). Due to the availability of the standard, the rate of quinoline-1-oxide formation could be quantified. The rate of quinoline-1-oxide formation in olfactory microsomes was 3-fold higher than that in hepatic microsomes (0.37 nmol/min/mg protein and 0.11 nmol/min/mg protein, respectively). The HPLC profiles of olfactory and hepatic microsome incubations without NADPH indicated that X4 formation induced by olfactory microsomes is CYP-independent. The addition of ABT, a general CYP inhibitor [22], resulted in dose-dependent inhibition of the quinoline metabolism catalyzed by olfactory microsomes (Fig. 2A). The IC50 values of ABT for inhibition of quinoline-1-oxide and quinoline-5,6-epoxide formation were 134 and 147 µM, respectively. ABT at the concentration of 400 µM decreased the metabolism of quinoline by approximately 80%. The olfactory metabolism of quinoline was not affected by BNPP, a specific inhibitor of carboxylesterases [23] (data not shown).

#### Metabolism of coumarin

**Figure 3:**
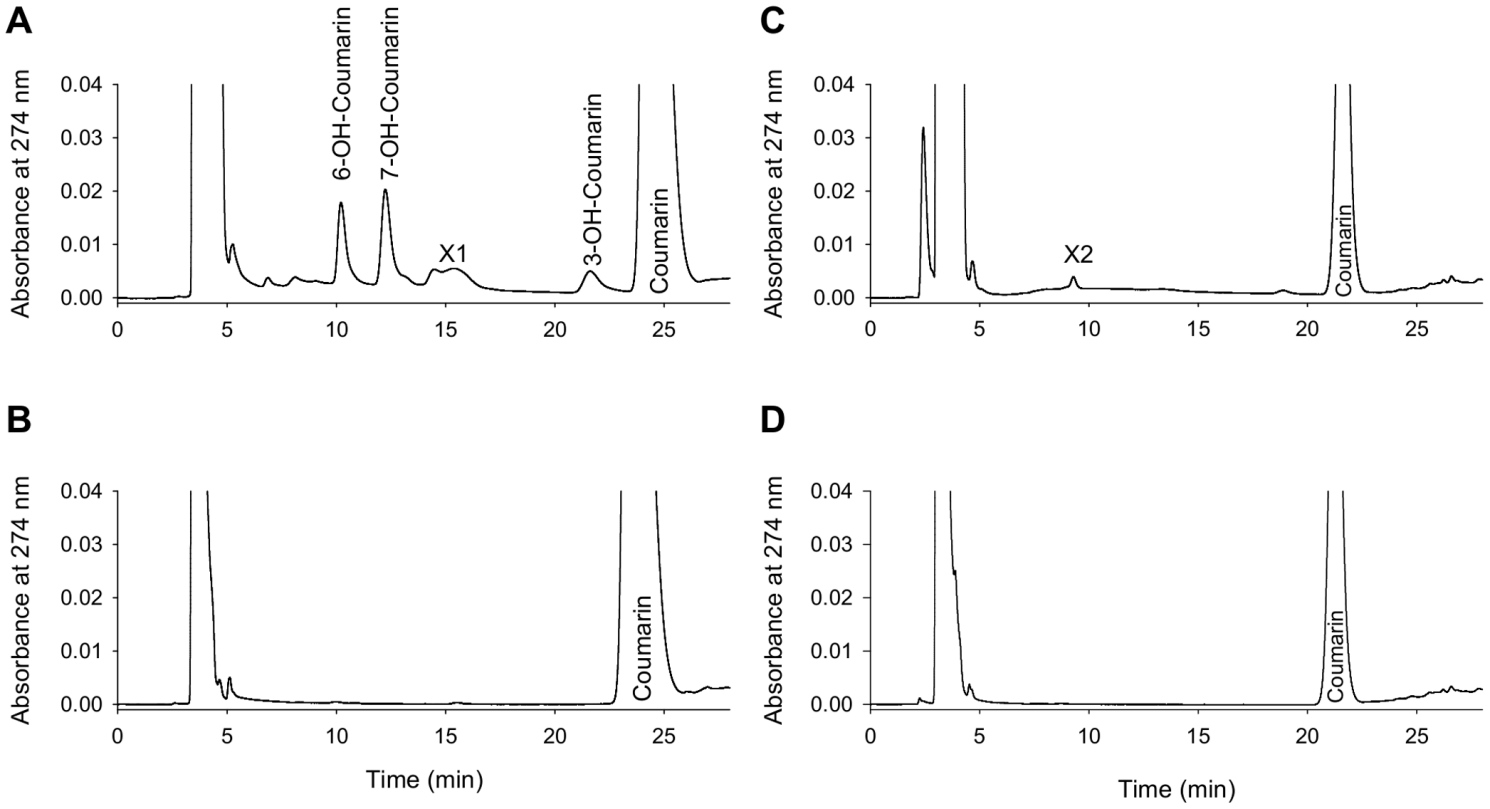
Representative HPLC profiles of coumarin metabolites formed *in vitro* by rat hepatic and olfactory microsomes. Four experimental conditions are shown: (A) reaction mixtures containing olfactory microsomes and NADPH; (B) reaction mixtures containing olfactory microsomes but no NADPH; (C) reaction mixtures containing hepatic microsomes and NADPH; (D) reaction mixtures containing hepatic microsomes but no NADPH.

Results from HPLC analysis of metabolites formed by incubating coumarin with olfactory or hepatic microsomes are shown in Fig. 3. Mass spectrometry analysis indicated that these compounds are oxygenated derivatives of coumarin (Fig. S4). After comparison of mass spectra with those of authentic standards (Fig. S5), we identified three main metabolites (7-hydroxycoumarin, 6-hydroxycoumarin and 3-hydroxycoumarin) in reactions involving olfactory microsomes (Fig. 3A). Low quantities of unidentifiable metabolites were also detected (peak X1). No metabolite was formed when NADPH was omitted (Fig. 3B). In contrast, only a small amount of one unidentifiable metabolite (peak X2) was formed when coumarin was incubated with hepatic microsomes (Fig. 3C). This metabolite was not found when NAPDH was omitted (Fig. 3D). The rate of coumarin biotransformation measured in OM incubation was 65-fold higher than that measured in liver incubation (13.3 nmol/min/mg protein and 0.2 nmol/min/mg protein, respectively). Furthermore, the addition of ABT to the olfactory microsome incubation inhibited formation of coumarin metabolites in a dose-dependent manner (Fig. 2B). ABT inhibited 3-hydroxy-, 6-hydroxy- and 7-hydroxycoumarin formation with IC50 values of 41, 52 and 19 µM, respectively. Ninety percent of metabolite formation was inhibited by 400 µM of ABT. However, coumarin metabolism was not affected by the addition of BNPP (data not shown).

#### Metabolism of isoamyl acetate

**Figure 4:**
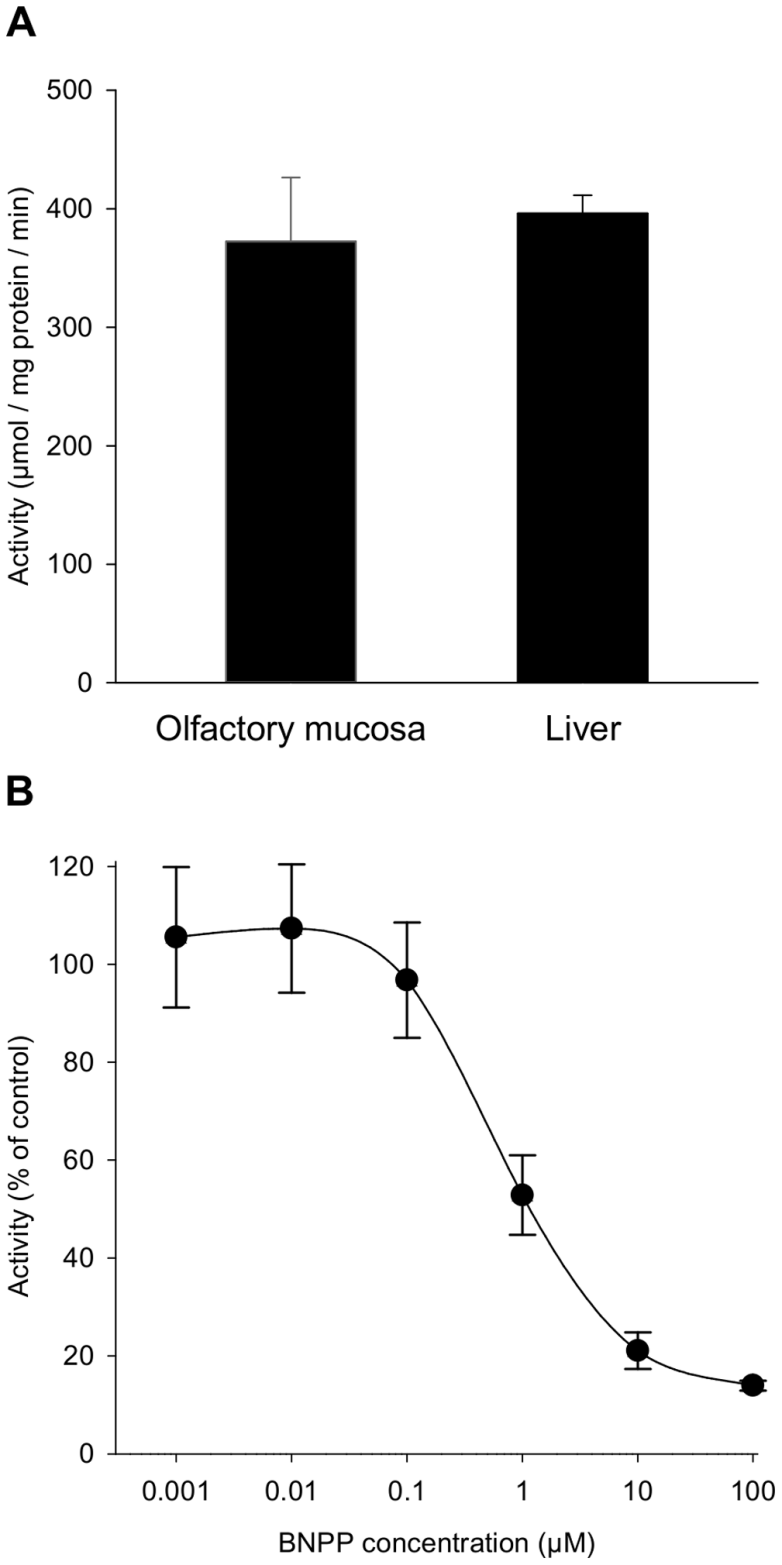
Hydrolysis of isoamyl acetate by olfactory and hepatic tissues and impact of the carboxylesterase inhibitor BNPP. (A) Hydrolase activity of rat olfactory and hepatic S9 fractions (mean ± S.E.M., n = 3 replicates); (B) inhibitory effect of BNPP on olfactory hydrolase activity (mean ± S.E.M., n = 3 replicates).

Carboxylesterases hydrolyze volatile esters to their corresponding acids [24]. Thus, we assessed the hydrolysis of isoamyl acetate by measuring acetic acid liberation in the reaction medium following incubation with olfactory or hepatic S9 fractions. Esterase activity was equivalent in both tissues (approximately 390 µmol acetic acid/mg protein/min) (Fig. 4A). Isoamyl acetate hydrolysis in the olfactory S9 fraction was inhibited in a dose-dependent manner by BNPP, a specific carboxylesterase inhibitor, with an IC50 value of 0.8 µM (Fig. 4B). In contrast, ABT had no effect on isoamyl acetate hydrolysis, which confirmed that this reaction was not dependent on the CYP enzymes (data not shown). These data indicate that carboxylesterases metabolize isoamyl acetate in the OM and liver and form acetic acid and isoamyl alcohol as metabolites.

### EOG Responses to Odorant Molecules and Odorant Molecule Derivatives

To assess the impact of biotransformation on the olfactory properties of the odorant molecules, we measured EOG responses to commercially available odorants and odorant metabolites. We also included other putative odorant derivatives (e.g., hydroxylated, methylated and glucurono-conjugated compounds) in these experiments. EOGs were recorded at concentrations of 1 µM, 10 µM and 100 µM.

**Figure 5:**
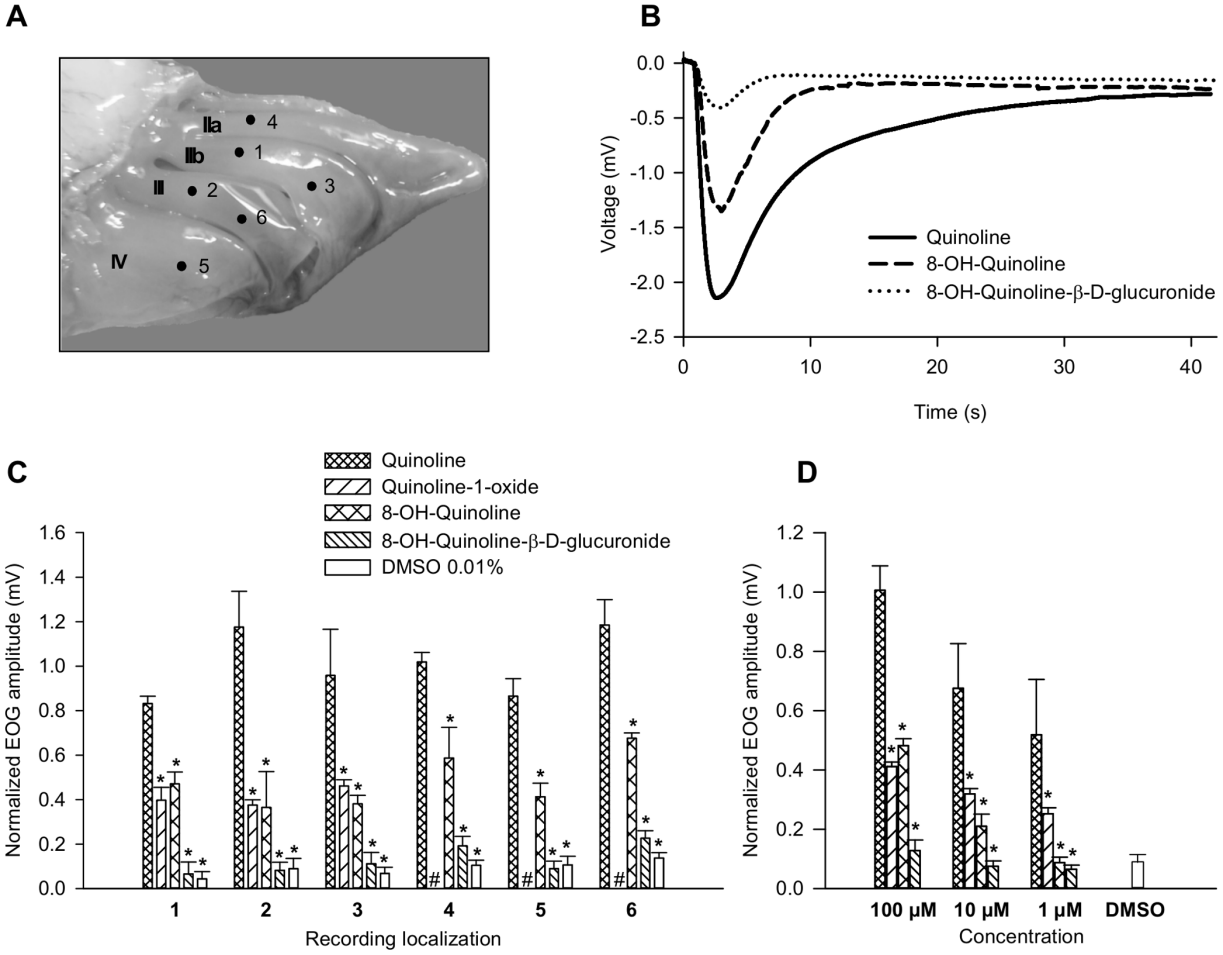
EOG response amplitudes elicited by quinoline and the quinoline derivatives. (A) Sagittal image of the rat olfactory system following sectioning of the head and removal of the nasal septum (roman numerals designate individual turbinates and arabic numerals indicate the sites at which odor responses were recorded). (B) Representative EOG recordings generated by quinoline and its derivatives (100 µM). (C) EOG response amplitudes recorded at different turbinate sites after stimulating the OM with quinoline and its derivatives (100 µM). The sign **#** indicates that EOG responses have not been recorded on these sites. (D) EOG response amplitudes induced by 1, 10 and 100 µM of quinoline and its derivatives (average of data recorded at different sites). Data are expressed as the mean ± S.E.M. (n = 4 rats). Asterisks (*) indicate significant differences between stimulation levels elicited by quinoline and the quinoline derivatives (Mann-Whitney test, p≤0.05).

The EOG responses induced by quinoline were compared to those induced by two hydroxylated metabolites, quinoline-1-oxide (Q-1-O) and 8-hydroxyquinoline (8-OH-Q), and the glucurono-conjugated metabolite 8-hydroxyquinoline-β-D-glucuronide (8-OH-Q-G). The EOG amplitudes were recorded at different locations on the olfactory epithelium (Fig. 5A) and normalized to IBMX responses. A representative EOG recording is shown in Fig. 5B. Regardless of the recording site, similar patterns of responses were observed when 100 µM of quinoline and quinoline derivatives were applied (Fig. 5C). The EOG responses to the two oxygenated products, 8-OH-Q and Q-1-O, was half of that elicited by quinoline. Glucuronide 8-OH-Q-G elicited a response equivalent to that induced by 0.01% dimethylsulfoxide (DMSO), which was used as vehicle. This result indicated that 8-OH-Q-G induced no olfactory response. At lower concentrations (1 and 10 µM), the compounds produced comparable response profiles but with lower amplitudes than those recorded at 100 µM (Fig. 5D).

**Figure 6:**
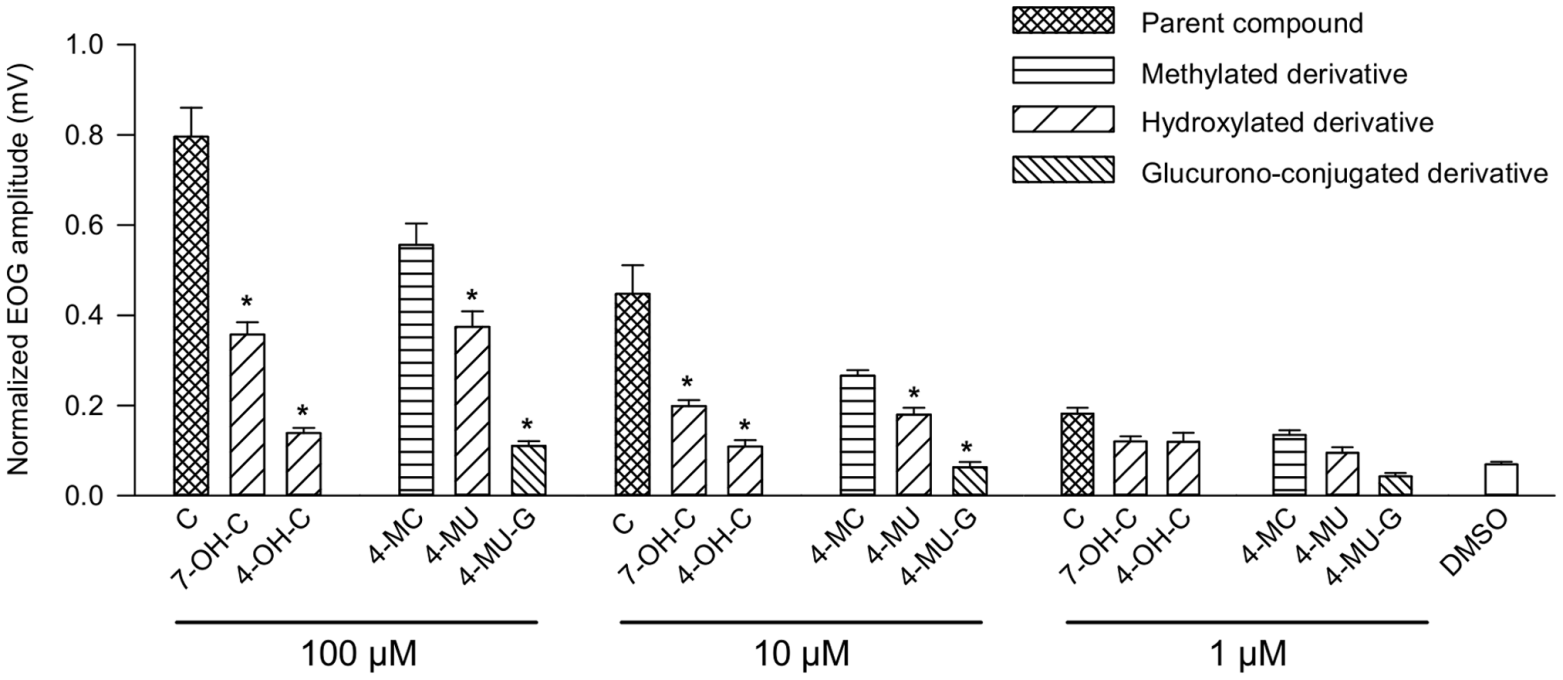
Normalized EOG maximum amplitudes elicited by 1, 10 and 100 µM of coumarin and the coumarin derivatives. Data are expressed as the means of data recorded at different sites ± S.E.M. (n = 4 rats). Asterisks (*) indicate significant differences between stimulations levels elicited by coumarin and coumarin derivatives (Mann-Whitney test, p≤0.05).

Similarly, the EOG responses to coumarin were compared to those induced by two hydroxylated metabolites, 4- and 7-hydroxycoumarin (4-OH-C and 7-OH-C), and the methylated derivative 4-methyl-coumarin (4-MC). The EOG responses to 4-MC were also compared to the responses elicited by the hydroxylated derivative 4-methylumbelliferone (4-MU) and to a glucurono-conjugated derivative of 4-MU, 4-methylumbelliferone-β-D-glucuronide (4-MU-G). The hydroxylated derivatives of coumarin (4-OH-C and 7-OH-C) and that of 4-MC (4-MU) elicited EOG amplitudes significantly lower than those induced by their parent compounds regardless of the applied concentration (Fig. 6). Furthermore, responses to 4-MC were lower in amplitude than the responses to coumarin. Low EOG amplitudes were also observed when the glucurono-conjugated derivative 4-MU-G was applied on the turbinates. The amplitudes of these responses were equivalent to those elicited by 0.01% DMSO. We therefore concluded that 4-MU-G did not elicit any measurable EOG response.

**Figure 7:**
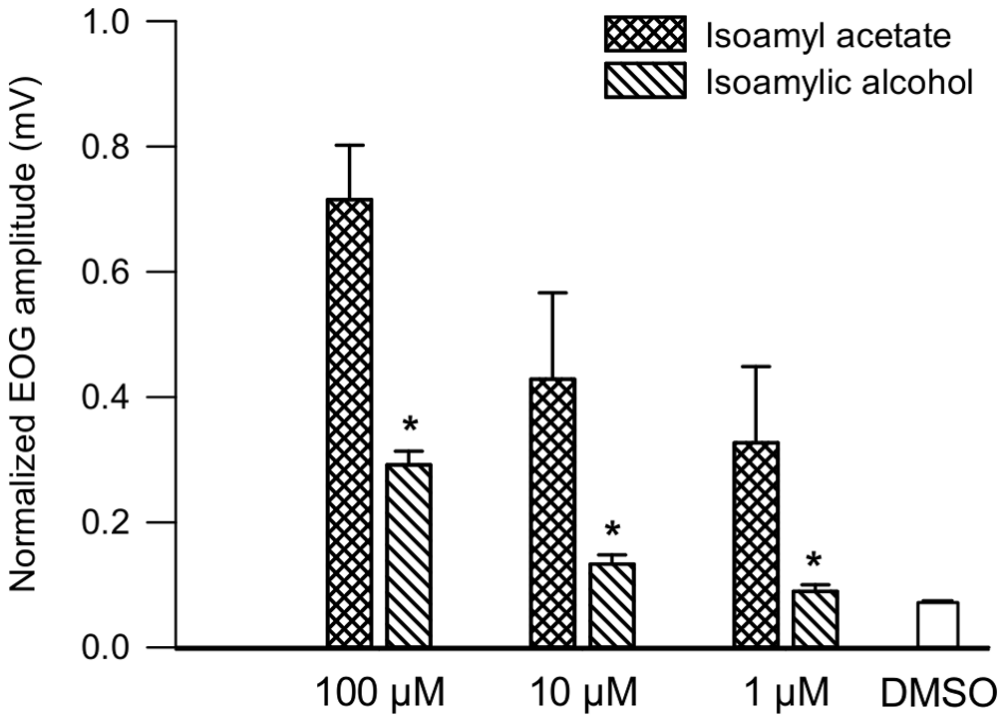
Normalized EOG maximum amplitudes elicited by 1, 10 and 100 µM of isoamyl acetate and isoamylic alcohol. Data are expressed as the mean of data recorded at different sites ± S.E.M. (n = 4 rats). Asterisks (*) indicate significant differences between the stimulation elicited by isoamyl acetate and isoamylic alcohol (Mann-Whitney test, p≤0.05).

Because the metabolism of isoamyl acetate creates isoamyl alcohol and acetic acid, we also measured EOG responses to each of these compounds. The results showed that the amplitudes of EOG responses to isoamyl alcohol were approximately 60% lower than those elicited by isoamyl acetate at any concentration (Fig. 7). Similar pattern of responses was recorded regardless the recording site Concerning acetic acid, we observed that repeated application of this compound on the OM caused tissue damage, making EOG recordings unreliable.

### Effects of XME Inhibitors on EOG Responses

To investigate the functional role of the olfactory XMEs, we recorded odorant-induced EOG responses before and after applying two phase I enzyme inhibitors, ABT and BNPP. First, we confirmed that superfusion of OM with ABT or BNPP was sufficient to inhibit CYP and carboxylesterase activities, respectively. For this purpose, the *in vitro* metabolism of quinoline, coumarin and isoamyl acetate was measured. All steps were performed as described previously except that the microsomal or S9 fractions were prepared from OM that had been superfused with inhibitors for 20 min. Superfusion of the OM with 400 µM of ABT or BNPP decreased the metabolism of quinoline/coumarin or isoamyl acetate, respectively, by approximately 30 to 80% (Fig. S6). In addition, we verified that the magnitude of 3-isobutyl-1-methylxanthine (IBMX)-induced EOG responses did not vary before and after inhibitor application to demonstrate that inhibitor treatment does not interfere with signal transduction in OSNs (data not shown).

**Figure 8:**
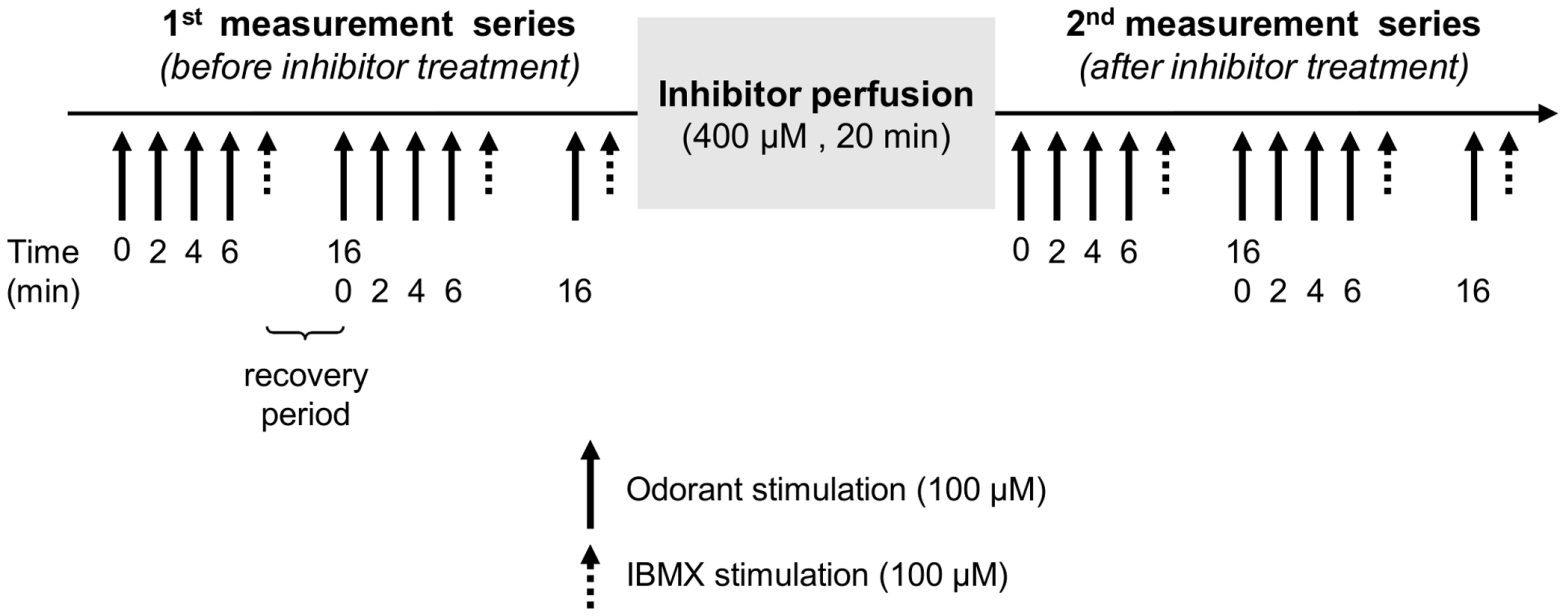
Schematic representation of the experimental protocol used to assess the effect of enzyme inhibitors (ABT and BNPP) on odorant-induced EOG responses.

**Figure 9:**
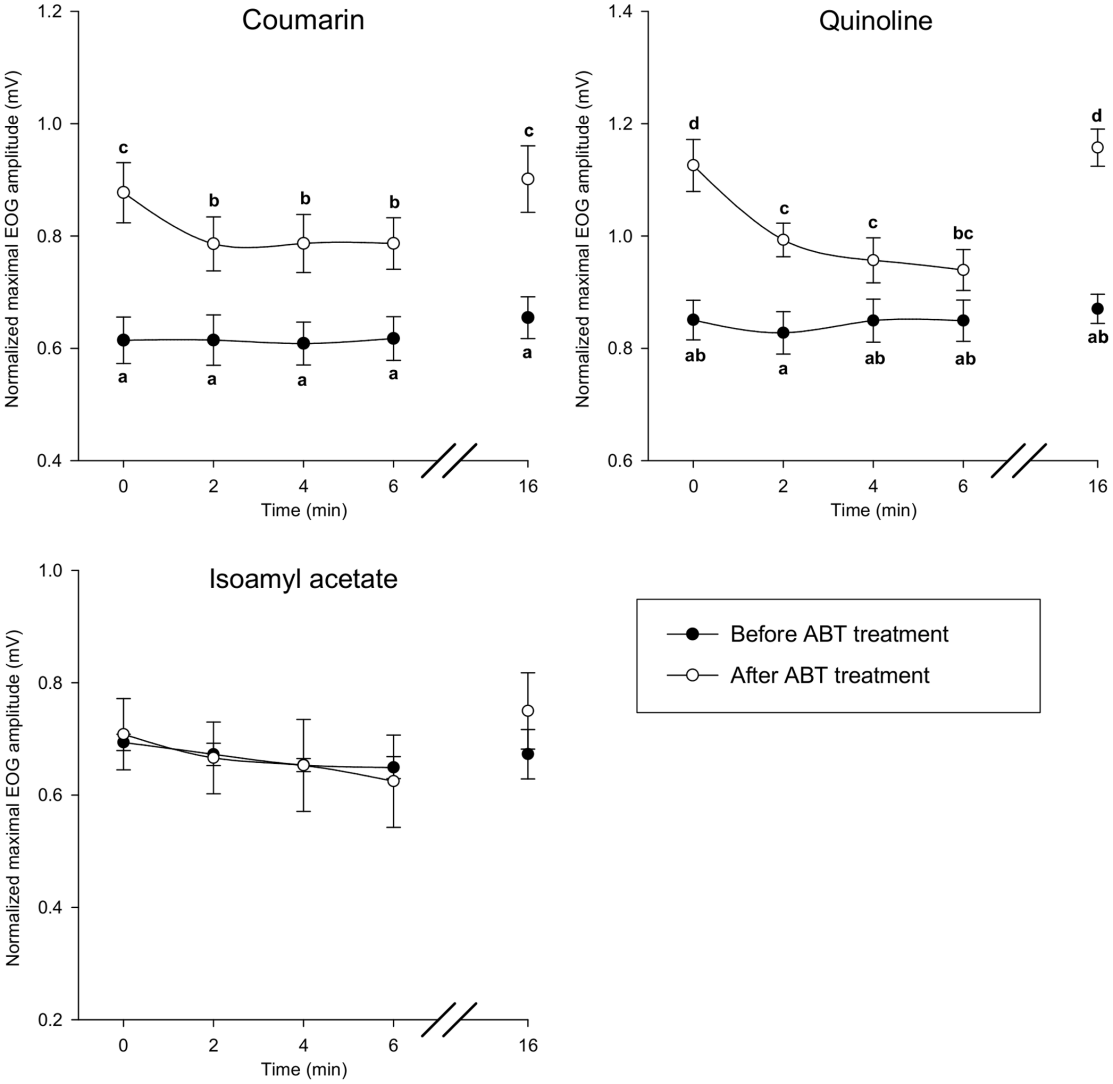
Time course of normalized EOG maximum amplitudes elicited by coumarin, quinoline and isoamyl acetate before and after ABT treatment (the protocol is presented in Fig. 8). EOG responses were recorded from the endoturbinate IIb. Data are expressed as the mean ± SEM (n = 5 rats). Data notated by distinct letters are significantly different (Bonferroni test, p≤0.05).

**Figure 10:**
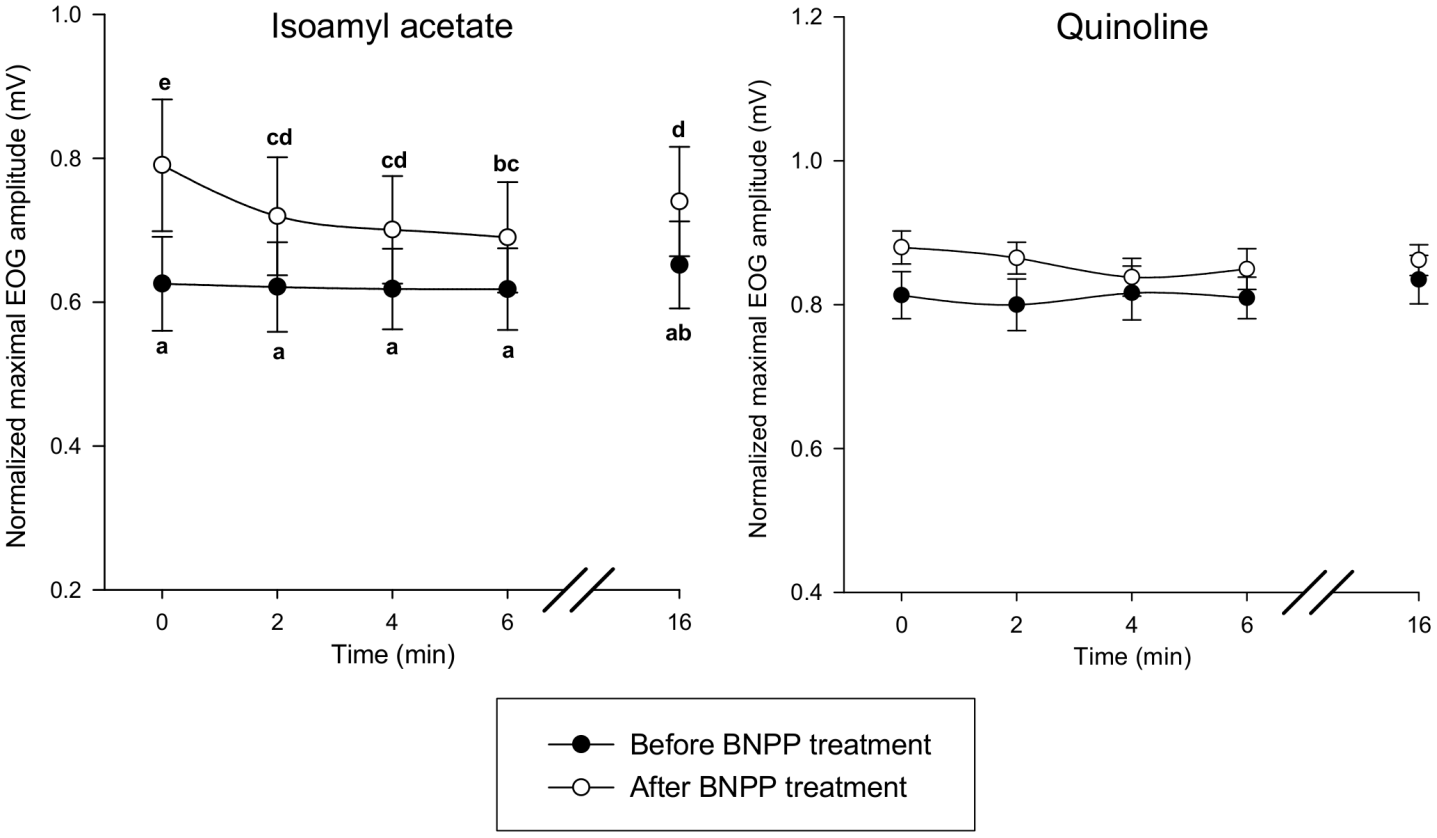
Time course of normalized EOG maximum amplitudes elicited by isoamyl acetate and quinoline and before and after BNPP treatment (the protocol is presented in Fig. 8). EOG responses were recorded from the endoturbinate IIb. Data are expressed as the mean ± SEM (n = 5 rats). Data notated by distinct letters are significantly different (Bonferroni test, p≤0.05).

The protocol presented in Fig. 8 was implemented to study the impact of enzyme inhibitors on EOG response. Before perfusion with ABT, we observed that successive stimulations by odorants at 2-min intervals produced identical EOG response amplitudes (Fig. 9). Treatment of the olfactory epithelium with ABT significantly increased the maximum EOG amplitudes elicited by coumarin and quinoline by 43% and 32%, respectively (Fig. 9). Interestingly, subsequent stimulations produced significantly lower EOG amplitudes. There was an 8-min refractory period before the same amplitude as that recorded just after ABT treatment could be recovered. Conversely, ABT treatment did not affect EOG responses to isoamyl acetate, a compound that is not metabolized by CYPs (Fig. 9). The EOG amplitudes elicited by isoamyl acetate before and after application of this inhibitor were identical. The onset and decay slopes of EOG recordings were stable over time and unaffected by ABT treatment (Fig. S7). Similarly, BNPP treatment significantly increased the maximal EOG response amplitude to isoamyl acetate by 30% (Fig. 10). As previously observed with ABT treatment, the EOG responses decreased when the OM was stimulated again. Further, we observed that the EOG response recovered to the initial levels following an 8-min refractory period. BNPP treatment did not modify the onset or decay slopes of EOG responses (Fig. S8). The EOG responses elicited by quinoline, which is not a carboxylesterase substrate, were not modified by BNPP treatment (Fig. 10).

## Discussion

The goal of this study was to determine whether odorant metabolism by olfactory tissue enzymes modulates the first step of olfactory perception. Because data on odorant biotransformation by the OM are scarce, we studied the phase I metabolism of several odorant molecules. Then, we evaluated the consequences of biotransformation on the stimulating properties of these odorants. We accomplished this step by comparing EOG responses to odorants and their derivatives and by measuring EOG responses following phase I enzyme inhibition. Collectively, these studies demonstrate that the OM can enzymatically modify odorant molecules and thus change their stimulating properties.

### OM has a High Odorant-metabolizing Capacity

*In vitro* metabolism studies have shown that the rat OM has the capacity to efficiently biotransform odorants such as quinoline, coumarin and isoamyl acetate. The extent of quinoline metabolism was higher in OM microsomes than in hepatic microsomes. Several hydroxylated metabolites, including quinoline-5,6-epoxide and quinoline-1-oxide, were formed following the phase I metabolism of quinoline. Similarly, the rate of coumarin metabolism in OM was much higher than the rate in the liver. This finding is consistent with previous studies [25]. The major metabolites formed by olfactory microsomes were 7-OH-coumarin and 6-OH-coumarin. The formation of o-hydrophenylacetaldehyde, a derivative of coumarin-3,4-epoxide, could not be measured in our experimental conditions because the detection of this ring-opened compound requires specific methods [26]. The strong decrease in metabolite formation observed when microsomes were pre-incubated with ABT suggests that CYPs are responsible for the metabolism of coumarin and quinoline. Previous reports have indicated that CYP2A3 and CYP2G1, which are highly expressed in rat OM, are involved in coumarin hydroxylation [25], [27]. The CYPs involved in the metabolism of quinoline in the OM are not known. However, quinoline is known to inhibit coumarin-7-hydroxylation, a reaction mainly catalyzed by CYP2A3 [28]. Thus, CYP2A3 also likely contributes to the biotransformation of quinoline in olfactory microsomes. In this study, furthermore, we showed that isoamyl acetate is readily metabolized by olfactory carboxylesterases. This result is in agreement with previous reports of high carboxylesterase activity in the OM [24], [29]. Taken together, these findings confirm that the rat OM displays remarkable odorant-metabolizing capacity. This property is closely related to high XME expression in the OM as previously reported by our group and others [7], [30]–[34].

### Odorants and their Derivatives Elicit Different Olfactory Responses

EOG responses to the hydroxylated derivatives of quinoline, coumarin and isoamyl acetate were significantly lower in amplitude than those elicited by the parent compounds. These results demonstrate that biotransformation modifies the olfactory-stimulating properties of odorants. It is generally thought that EOG amplitudes represent the sum of generator potentials created by individual OSNs in the recording field [35]. Changes in EOG amplitude can therefore result from variation in the number of activated OSNs (i.e., the number of activated ORs) or from variation in the intensities of individual neuron responses. Recently, using a heterologous expression system to study the activation profiles of approximately 460 mammalian ORs, Saito et al. showed that coumarin activated 20 ORs whereas 4-hydroxycoumarin only activated one OR, mOR41-1 [36]. Though this receptor was activated by both compounds, coumarin was more potent than its hydroxylated derivative. These data are in agreement with our observations, indicating that odorant hydroxylation can reduce both the numbers of activated OSNs and their individual response intensities. It is nevertheless difficult to generalize this finding. Indeed, a number of odorants possessing a hydroxyl group have been shown to efficiently activate ORs [36], [37]. In some cases, the presence of a hydroxyl group seems to even be essential for agonist activity. For example, the tertiary alcohol moiety of lyral is primarily responsible for activating the murine receptor MOR23 and generating an olfactory response [38]. A functional study of the mouse receptor mOR-EG also demonstrated the importance of a hydroxyl group attached to the benzene ring of odorants in activating this receptor [39]. This hydroxyl group forms a hydrogen bound with a specific amino acid in the receptor and is essential for ligand binding.

In addition to the impact of hydroxylation, we also examined the consequence of glucuronidation on olfactory properties of odorants. Indeed, significant glucuronidation activities have been reported in the rat OM [7], [15]. In particular, we observed that glucuronidation of the coumarin derivative 4-MU is higher in the OM than in the liver, indicating that the OM has the capacity to glucurono-conjugate efficiently coumarin metabolites [7]. In the present study, we demonstrated that the glucurono-conjugated metabolites of quinoline and coumarin were unable to elicit EOG responses. This finding is in accordance with a previous study showing that olfactory cAMP production *in vitro* is stimulated by parent odorants but not their glucuronidated derivatives [14]. Mitral cell response is also low in rats exposed to odorants that are efficiently glucurono-conjugated by the OM [15]. The rodent OM predominantly expresses the UGT2A1 isoform, but the UGT2A2 and UGT1A6 isoforms have also been detected [7], [15], [40]. UGT2A1 is expressed in sustentacular cells, Bowman glands and, interestingly, in olfactory sensory cilia [14], [40], [41]. This expression pattern supports the hypothesis that olfactory UGTs are involved in the extinction of olfactory signals. Indeed, the conjugation of odorants with large hydrophilic entities such as glucuronic acid increases steric hindrance and therefore may hinder interactions with OR.

### Mucosal XMEs Modulate Peripheral Olfactory Responses

As we observed that biotransformation of odorants occurs significantly in OM, we postulated that this process could affect the olfactory responses. If this is the case, this would mean that the amplitudes of EOG responses to odorants, but also perhaps to their metabolites, that have been recorded take into account the impact of biotransformation processes. As EOG amplitudes elicited by metabolites are lower than those induced by the parent compounds, this would result finally in a decrease in EOG amplitudes. Similarly, it could also be assumed that metabolic reactions can modulate responses elicited by hydroxylated metabolites since numerous phase 2 enzymes (such as UGTs) are expressed in the olfactory mucosa. Therefore, to assess the influence of peripheral XMEs on olfactory responses, we compared EOG responses before and after treatment of the OM with ABT and BNPP, two inhibitors that irreversibly inactivate CYPs and carboxylesterases, respectively [22], [23]. ABT treatment of the olfactory epithelium increased EOG responses elicited by quinoline and coumarin (which are metabolized by CYPs) but did not modify the EOG response to isoamyl acetate. Conversely, BNPP treatment modified EOG responses to isoamyl acetate (which is metabolized by carboxylesterases) but not those elicited by quinoline and coumarin. These observations indicate that the inhibitors do not affect OSN receptor-transduction mechanisms but do target enzymes (CYPs and carboxyl esterases) that modulate olfactory responses. The increase in EOG responses observed following inhibitor application may result from an increased number of recruited receptors or from modification of odorant affinity for olfactory receptors. Furthermore, the kinetics of the EOG responses were not affected by ABT or BNPP, indicating that these treatments did not perturb the OSN environment. However, a slight decrease in EOG amplitude was observed when OM was stimulated repeatedly with odorants after inhibitor treatment. This effect could be a consequence of neuronal adaptation that occurs when OSNs are continuously exposed to odorants to prevent saturation of the cellular transduction machinery [42]. We speculate that XME inhibition drastically slows the metabolism of odorants, thereby prolonging the presence these molecules in the perireceptor space and inducing OSN adaptation. XMEs may therefore participate in maintaining olfactory system sensitivity.

Because enzymes localized to the nasal mucus can also convert odorants such as vanillin [5], it cannot be ruled out that the mucus enzymes had an impact on our study. However, previous observations showed that washing the olfactory epithelium with Ringer buffer strongly increased the response amplitude of olfactory bulb glomerulus to vanillin, indicating that a large portion of the mucus was removed by washing [43]. Therefore the enzymes targeted by the inhibitors are likely localized in the OM. XMEs have been detected in different cell types, including sustentacular cells and the cilia of OSNs [10], [44]. XMEs are classically found inside cells, mostly in the endoplasmic reticulum and in the cytosol. This localization seems to be incompatible with a fast metabolism of odorant molecules. A possibility might be that odorants could be biotransformed by enzymes present in the plasma membrane of cells. Indeed, a number of studies reported the presence of CYPs at the extracellular face of the plasma membrane of different mammal cell types (for a review, see [45]). These studies also demonstrated that these membrane enzymes were catalytically active, suggesting they might play a role in the metabolism of xenobiotics. The presence of active CYPs, and perhaps other XMEs, at the cell surface of olfactory cells, close to the receptors, could have an impact both on the intensity and the quality of olfactory signals. This assumption is supported by a recent study that reported detection of odorant metabolites in the exhaled air following odorant inhalation in human subjects [46]. In the same study, the inhalation of a CYP inhibitor with a ketone odorant modified the odor quality perceived by the participants, indicating that metabolites formed by CYPs contributed to the ketone odor [46]. These findings support the idea of a fast metabolic process and indicate that metabolites formed by olfactory XMEs, particularly those issued from CYP-catalyzed reactions, can participate in olfactory coding and thus noticeably affect odor quality.

In conclusion, the present study demonstrates that the olfactory mucosa has the capacity to efficiently metabolize odorant molecules and that odorant metabolism can modulate the intensity of olfactory responses. Our findings, together with the observations made by Nagashima et al. [5], support the idea that enzymes in the environment surrounding olfactory receptors, i.e., in the olfactory mucosa and mucus, shape olfactory perception by modulating both the intensity and quality of odorant signals. Evaluating the contribution of different metabolic events in this process would enhance our understanding of peripheral chemosensory mechanisms.

## Materials and Methods

### Chemicals

**Figure 11:**
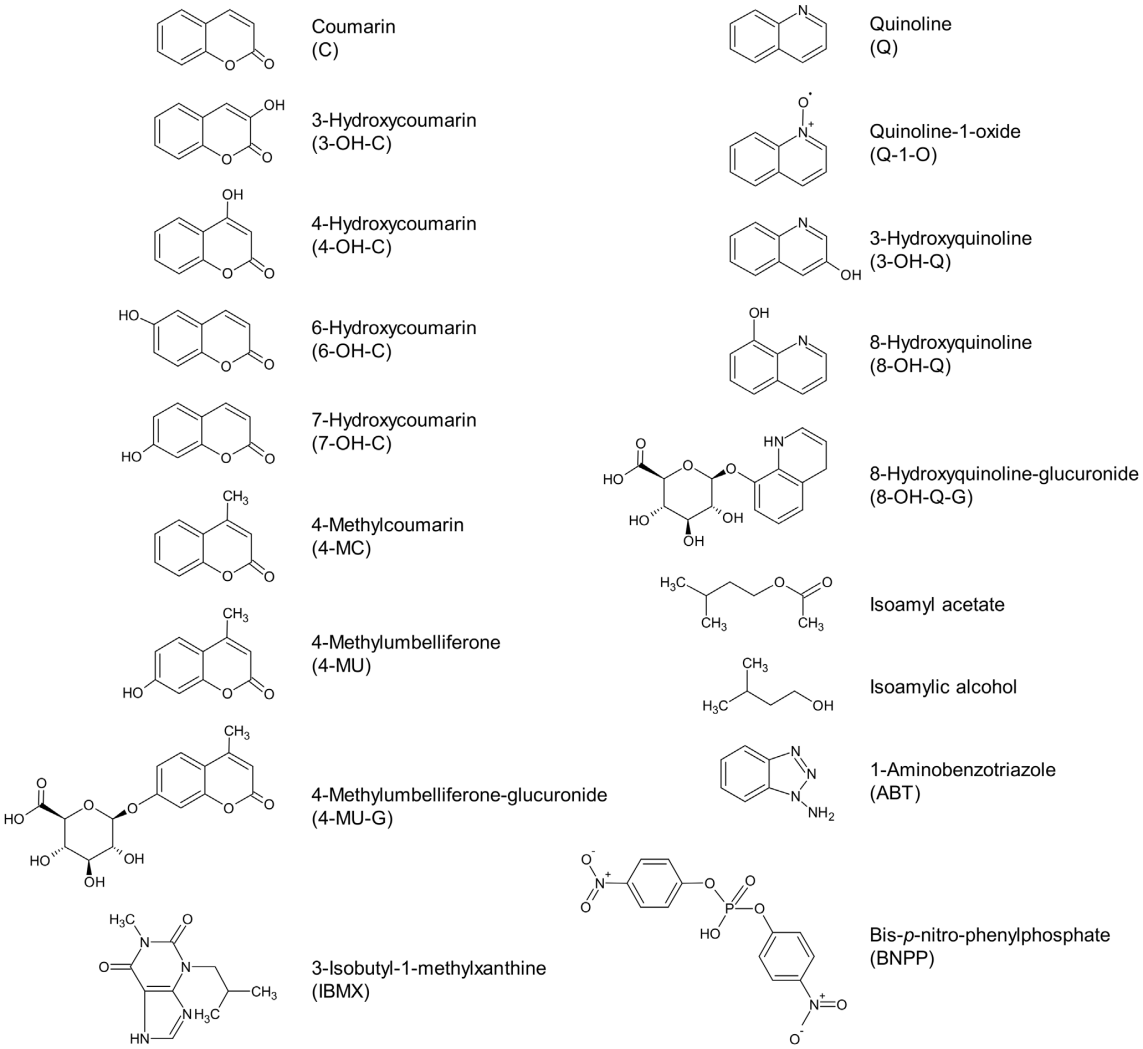
Chemical structures and abbreviations of molecules used in the study.

The odorant molecules, their derivatives and the enzyme inhibitors used in this study were purchased from Sigma-Aldrich (Saint-Quentin Fallavier, France), Interchim (Montluçon, France) or Merck (Fontenay sous Bois, France). The chemical structures and molecule characteristics of these compounds are presented in Fig. 11 and Table S1, respectively. All compounds were of the highest available quality.

### Ethics Statement

The experiments were carried out in accordance with the French Ministry of Agriculture guidelines for the care and use of laboratory animals. The experimental protocol was approved by the local animal ethics committee of the University of Burgundy, Dijon, France (approval number EXT0109).

### Animals

Seven-week-old male Wistar rats were purchased from Janvier (Le Genest Saint Isle, France). Animals were housed in temperature- (20–22°C) and relative humidity-controlled (30–70%) conditions with a 12-h light/dark cycle. Animals had free access to water and A04-10 food pellets (Safe, Augy, France).

### Metabolism of Odorant Molecules

#### Preparation of microsomal and S9 fractions

Animals were sacrificed by decapitation. Livers and OM were immediately removed and snap-frozen in liquid nitrogen. OM included both endoturbinates I to IV and ectoturbinates 1 to 4, according to the nomenclature of Ressler et al. [47]. Samples were stored at −80°C until they were differentially centrifuged to obtain microsomes and S9 fractions. Pools of five OM were homogenized in 2 mL of 50 mM Tris-HCl buffer (pH 7.5) containing 0.25 M sucrose and 1 mM EDTA using an Ultra-Turrax homogenizer. The homogenate was centrifuged twice at 1500 g for 10 min to discard debris. The supernatant fraction was then centrifuged at 8500 g for 10 min then at 14 500 g for 20 min. The supernatant fraction obtained following this step was designated the S9 fraction. To obtain microsomes, the S9 fraction was centrifuged at 105 000 g for 60 min. The resulting pellet was resuspended in homogenization buffer. Hepatic subcellular fractions were prepared as described previously [48]. Microsomes and S9 fractions were stored in small aliquots at −80°C until use. The protein levels of these fractions were quantified by the method of Bradford [49] using bovine serum albumin as a standard.

#### Metabolism assays

Incubations of odorants (coumarin, quinoline and isoamyl acetate) with subcellular fractions were carried out as described in Table S2. Coumarin and quinoline were incubated with microsomes in order to investigate the ability of enzymes localized in the endoplasmic reticulum (mainly CYPs) to metabolize these odorants. As the mammalian carboxylesterases are localized in the endoplasmic reticulum and in the cytosol of cells [50], isoamyl acetate biotransformation was examined by incubating this compound with S9 fraction. Reactions were performed in triplicate at 37°C and were initiated by adding odorant to the mixtures after a 5-min pre-incubation period. Reactions involving coumarin and quinoline were carried out for 30 min and 90 min, respectively, and were terminated by adding 100 µl of ice-cold acetonitrile. Subsequently, the products were centrifuged at 12 000 g for 10 min. An aliquot of the supernatant (100 µl) was analyzed by reverse phase high performance liquid chromatography (HPLC) using a Waters HPLC system (Milford, MA, USA) equipped with a pump (model 600), an autosampler thermostat set at 10°C (model a717 plus), a photodiode array UV detector (model 996) and an Uptisphere C18-ODB column (5-µm particle size, 150×4.6 mm; Interchim, Montluçon, France). The effluent from the column was routed to an UV detector, which can scan wavelengths from 210 to 400 nm. The solvent gradient program used to separate quinoline and its metabolites was set at 5% methanol in water for 5 min followed by a linear gradient of methanol increasing to 40% in 15 min (at 1 mL/min). The separation of coumarin and its metabolites was achieved using a solvent gradient program (at 0.6 mL/min) set at: 25% acetonitrile/75% formic acid (0.01% in water) for 10 min, a linear gradient to 80% acetonitrile for 20 min, hold at 80% acetonitrile for 10 min, and then return to the initial conditions. Additionnal liquid chromatography - mass spectrometry analyses were carried out to identify quinoline and coumarin metabolites. Details about instrumentation and results are given as supplemental information (Text S1).

Reactions involving isoamyl acetate were carried out for 10 min and were terminated by heating at 80°C for 15 min. After centrifugation at 12000 g for 10 min, isoamyl acetate metabolism was assessed by quantifying the acetate in a sample volume of 100 µL using an acetic acid assay kit (Megazyme, Bray, Ireland).

### Electrophysiological Studies

#### Stimuli solution preparation

Odorant stock solutions (1 M) were prepared in DMSO. Working solutions were prepared extemporaneously by diluting stock solutions with the Ringer solution. The final concentration of DMSO in odorant solutions did not exceed 0.01%. The IBMX stock solution (10 mM) was also prepared in DMSO. This stock solution was diluted 100-fold with the Ringer solution to produce a working solution (100 µM) containing 0.1% DMSO.

#### Electroolfactogram recordings

To assess sensory responses induced by odorant stimulation of the olfactory mucosa, we recorded EOGs. This method measures the summated, odorant-induced generator potentials of the OSNs on the olfactory epithelium surface [35]. We used a submerged EOG technique because it allowed us to quantify responses elicited by non-volatile and low-volatility compounds and to deliver pharmacological agents [51]. Rats were sacrificed by CO_2_ exposure and decapitated. The head was hemisected and the nasal septum was carefully removed to expose the endoturbinate system of the main olfactory system. The tissue was then superfused continuously (5 mL/min) at room temperature (22–25°C) with modified Ringer buffer containing 140 mM NaCl, 5 mM KCl, 2 mM CaCl_2_, 2 mM MgCl_2_, 10 mM HEPES [4-(2-hydroxyethyl)-1-piperazineethanesulfonic acid], adjusted to pH 7.2 and to 320 mOsm.L^−1^ with glucose. Odorant stimuli were applied to the turbinates using a custom-built odorant delivery system consisting of a Rheodyne six-port injection valve equipped with a 200-µL loop. This valve injects odorant into the continuous stream of the Ringer solution. The distal part of the delivery system was fixed to a micromanipulator and adjusted to produce uniform flow in the vicinity of the recording electrode. The recording electrode was an Ag/AgCl wire positioned in a disposable pipette tip filled with a conductive gel (WPI, Sarasota, USA). The reference electrode was an 8-mm diameter Ag/AgCl disk covered with conductive gel that was placed under the rat hemi-head. Electrical signals were amplified using an AC/DC differential amplifier (A-M Systems, model 3000, Phymep, Paris, France), run through a low-pass filter of 300 Hz, digitized at 100 Hz using a Digidata 1440A interface board (Axon Instruments, DIPSI Industrie, Châtillon, France) and acquired using Axoscope 10.2 software (Axon Instruments). The data were analyzed using Clampfit software (Axon Instruments). The peak amplitudes of EOG responses were measured at the maximum negative voltage deflection from baseline. The data were normalized by dividing the peak amplitude by the maximum amplitude elicited by 100 µM of IBMX, a phosphodiesterase inhibitor that activates the transduction pathway bypassing the receptor step.

The protocol used to assess the effect of XME inhibitors (ABT or BNPP) on EOG responses is presented in Fig. 8. First, two series of odorant stimulations at 2-min intervals were performed with an 8-min recovery period between each series. Under control conditions, a 2-min interval is sufficient to recover normal response amplitude. Then, the inhibitor was diluted in the Ringer buffer (final concentration 400 µM) and applied to the olfactory turbinates at a rate of 2.5 mL/min for 20 min using the Rheodyne injection valve. The preparation was rinsed for 5 min with the Ringer buffer and the epithelium was stimulated by applying odorant using the same procedures as described above. EOG responses were recorded on endoturbinate IIb. IBMX was applied at the end of each measurement series for data normalization as described above. In addition to the peak maximum amplitude, the onset slope (10% to 90%) and the fast decay slope (90% to 40%) were measured and normalized to the corresponding EOG peak amplitude.

### Statistical Analysis

The non-parametric Mann-Whitney test was applied to assess differences between the EOG response amplitudes induced by the odorants and those induced by their derivatives. A two-way repeated-measures ANOVA was used to analyze the data from the EOG experiments involving enzyme inhibitors. When the ANOVA revealed a statistically significant effect, the Bonferroni post-hoc test was applied to compare EOG responses recorded throughout the protocol. The results were considered to be significant if p<0.05. All statistical analyses were carried out using Statistica software (version 8; StatSoft, Maisons-Alfort, France).

## Supporting Information

Figure S1 LC-ESI-MS/MS chromatograms (mode scan products of molecular ion [M+H]+) and UV-chromatogram (at 290 nm) of a mixture of authentic standards (quinoline and quinoline-1-oxide) (A) and of metabolites formed after incubation of quinoline with rat olfactory microsomes (B).
(TIF)

Figure S2 Product ion spectra of the molecular ions [M+H]+ at m/z 146 of authentic standard of quinoline-1-oxide (A) and compounds formed after incubation of quinoline with rat olfactory microsomes (B).
(TIF)

Figure S3 Product ion spectra of the molecular ions [M+H]+ of quinoline-derivated compounds at m/z 162.
(TIF)

Figure S4 LC-ESI-MS/MS chromatogram (mode scan products of molecular ion [M-H]- at m/z 161) and UV-chromatogram (at 274 nm) of a mixture of authentic standards (coumarin, 7-hydroxycoumarin, 6-hydroxycoumarin and 3-hydroxycoumarin) (A) and of metabolites formed after incubation of coumarin with rat olfactory microsomes (B).
(TIF)

Figure S5 Product ion spectra of the molecular ions [M-H]- at m/z 161 of authentic standards of coumarin metabolites (A) and compounds formed after incubation of coumarin with rat olfactory microsomes (B).
(TIF)

Figure S6 Effect of superfusion of the olfactory mucosa with 400 µM ABT on quinoline (A) and coumarin (B) metabolite formation and effect of superfusion with 400 µM BNPP on carboxylesterase activity (C). Values represent mean of 3 replicates ± S.E.M.
(TIF)

Figure S7 EOG onset (A) and decay (B) slopes elicited by coumarin, quinoline and isoamyl acetate before and following ABT treatment. Onset slopes (10% to 90%) and fast decay slopes (90% to 40%) were normalized to the corresponding EOG peak amplitude. Data are expressed as the mean ± SEM (n = 5 rats). No comparison was significantly different (Bonferroni test, p≤0.05).
(TIF)

Figure S8 EOG onset (A) and decay (B) slopes elicited by isoamyl acetate and quinoline and before and after BNPP treatment. Onset slopes (10% to 90%) and fast decay slopes (90% to 40%) were normalized to the corresponding EOG peak amplitude. Data are expressed as the mean ± SEM (n = 5 rats). No comparison was significantly different (Bonferroni test, p≤0.05).
(TIF)

Table S1 Characteristics of molecules used in the study.
(PDF)

Table S2 Incubation conditions used to study the *in vitro* metabolism of odorants.
(PDF)

Text S1 Liquid chromatography - mass spectrometry analyses
(PDF)
