# Supplementary figures and images for: Odorant Metabolism Catalyzed by Olfactory Mucosal Enzymes Influences Peripheral Olfactory Responses in Rats
Source: PLoS One. 2013 Mar 26;8(3):e59547. doi: 10.1371/journal.pone.0059547 (PMC3608737; doi:10.1371/journal.pone.0059547)

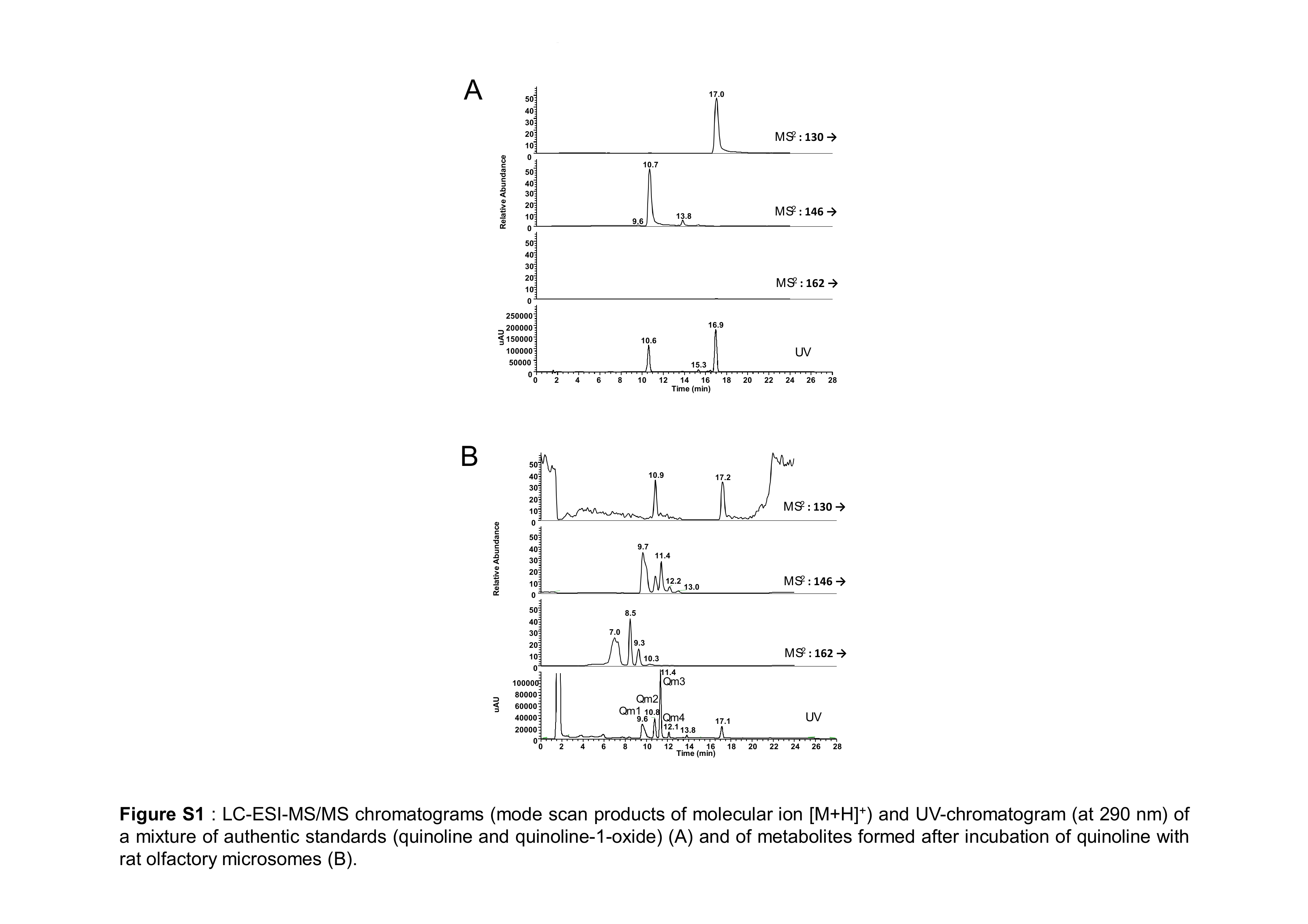

Supplement: Figure S1 — LC-ESI-MS/MS chromatograms (mode scan products of molecular ion [M+H]+) and UV-chromatogram (at 290 nm) of a mixture of authentic standards (quinoline and quinoline-1-oxide) (A) and of metabolites formed after incubation of quinoline with rat olfactory microsomes (B). (TIF) [file pone.0059547.s001.tif]

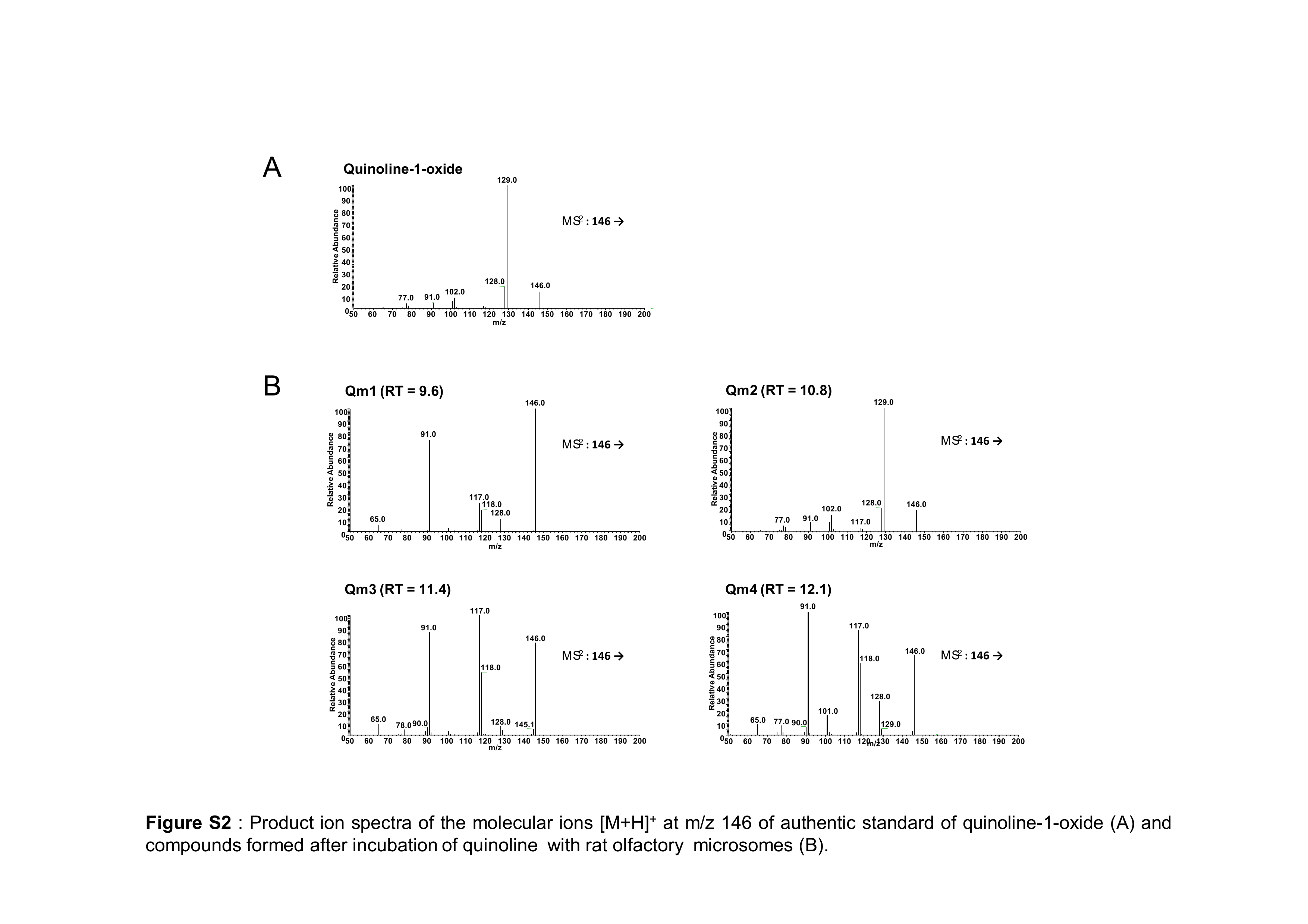

Supplement: Figure S2 — Product ion spectra of the molecular ions [M+H]+ at m/z 146 of authentic standard of quinoline-1-oxide (A) and compounds formed after incubation of quinoline with rat olfactory microsomes (B). (TIF) [file pone.0059547.s002.tif]

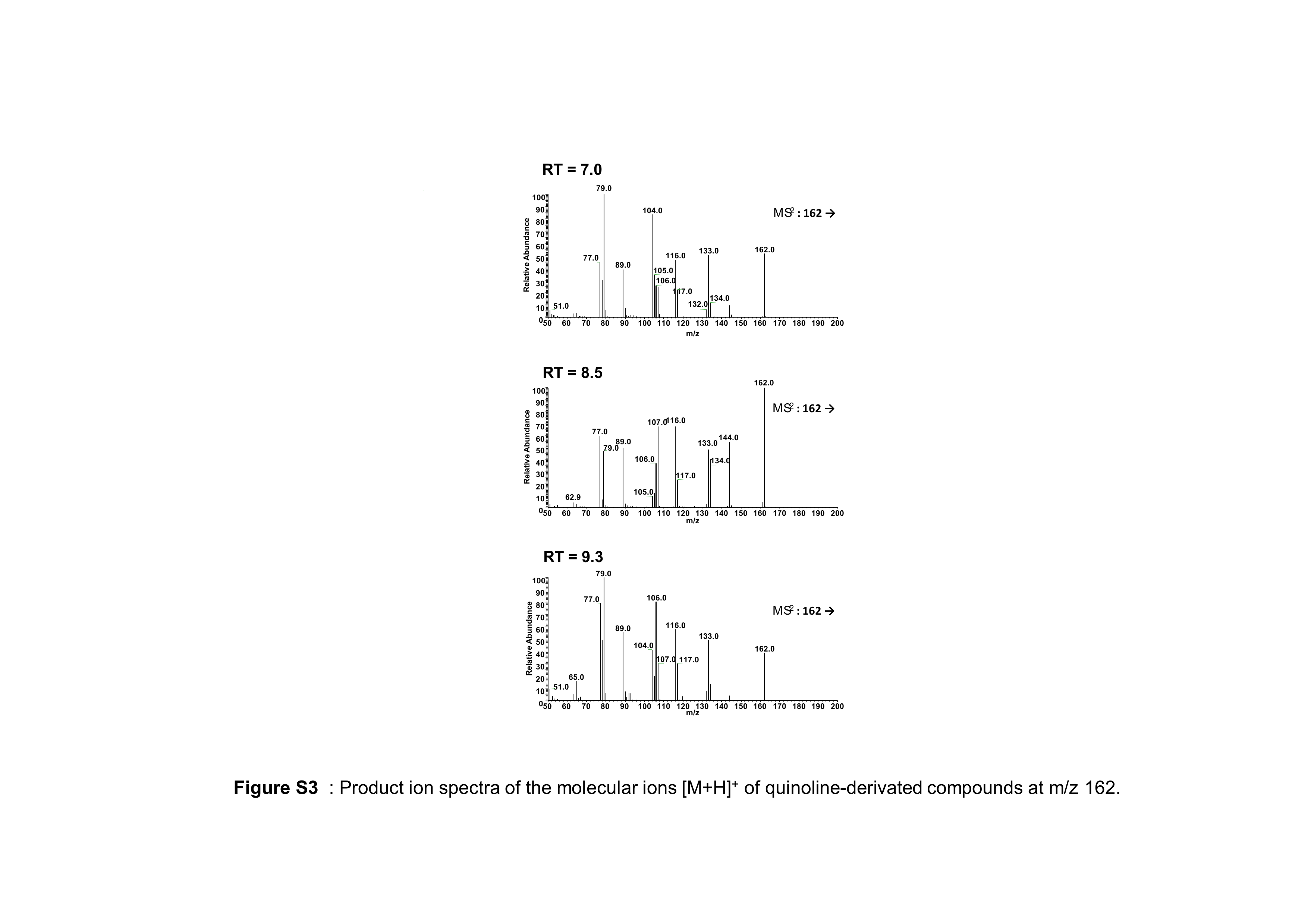

Supplement: Figure S3 — Product ion spectra of the molecular ions [M+H]+ of quinoline-derivated compounds at m/z 162. (TIF) [file pone.0059547.s003.tif]

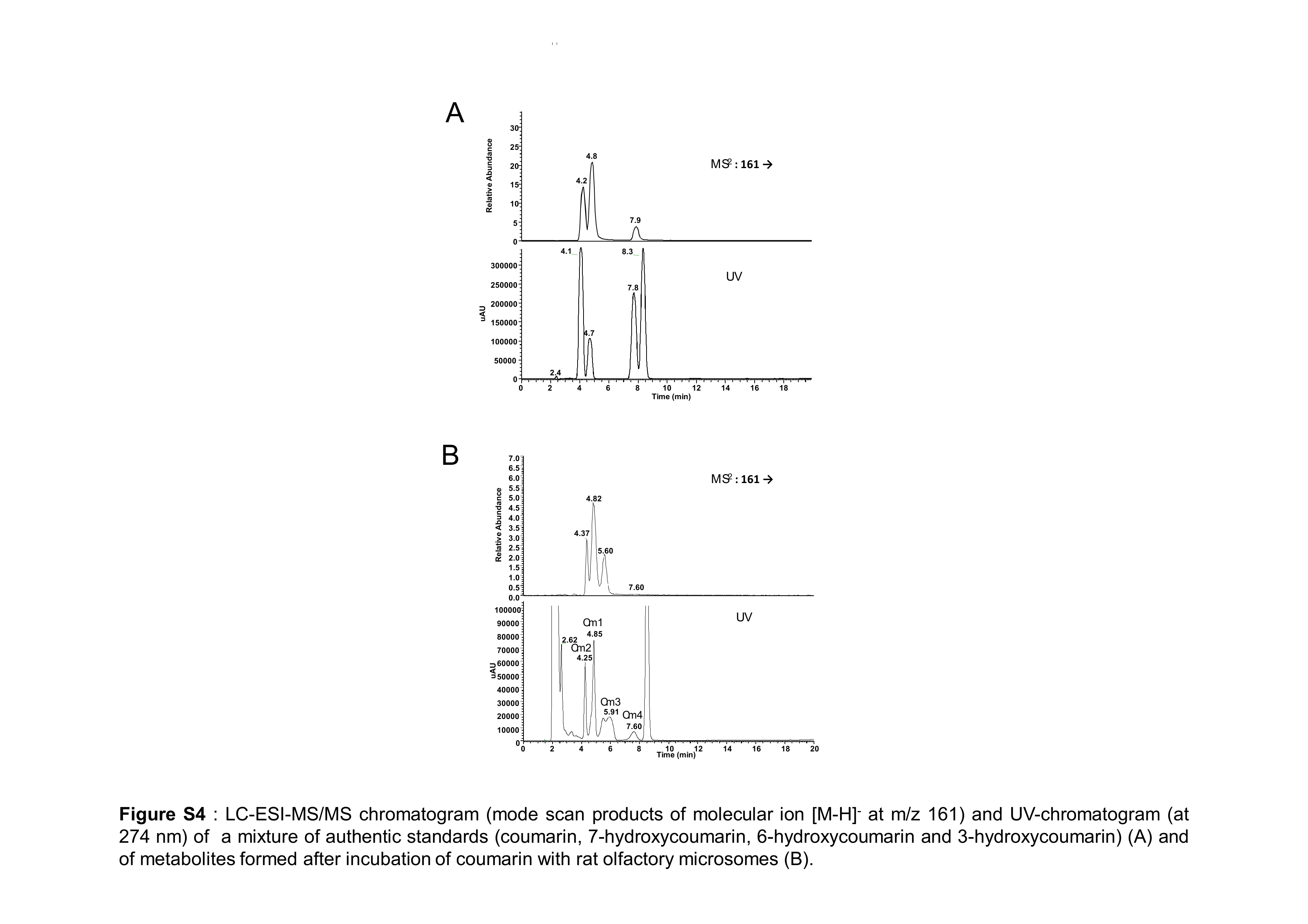

Supplement: Figure S4 — LC-ESI-MS/MS chromatogram (mode scan products of molecular ion [M-H]- at m/z 161) and UV-chromatogram (at 274 nm) of a mixture of authentic standards (coumarin, 7-hydroxycoumarin, 6-hydroxycoumarin and 3-hydroxycoumarin) (A) and of metabolites formed after incubation of coumarin with rat olfactory microsomes (B). (TIF) [file pone.0059547.s004.tif]

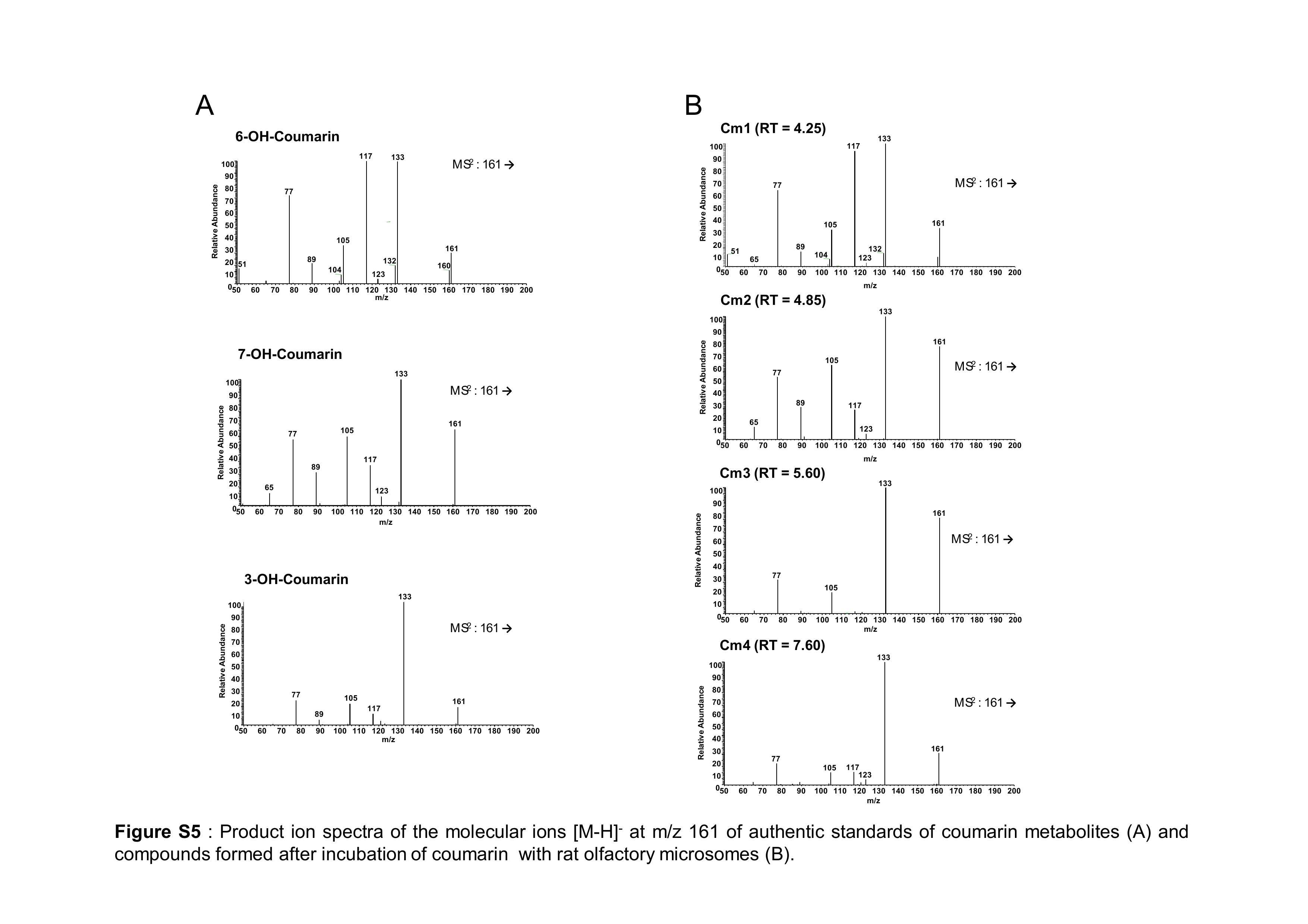

Supplement: Figure S5 — Product ion spectra of the molecular ions [M-H]- at m/z 161 of authentic standards of coumarin metabolites (A) and compounds formed after incubation of coumarin with rat olfactory microsomes (B). (TIF) [file pone.0059547.s005.tif]

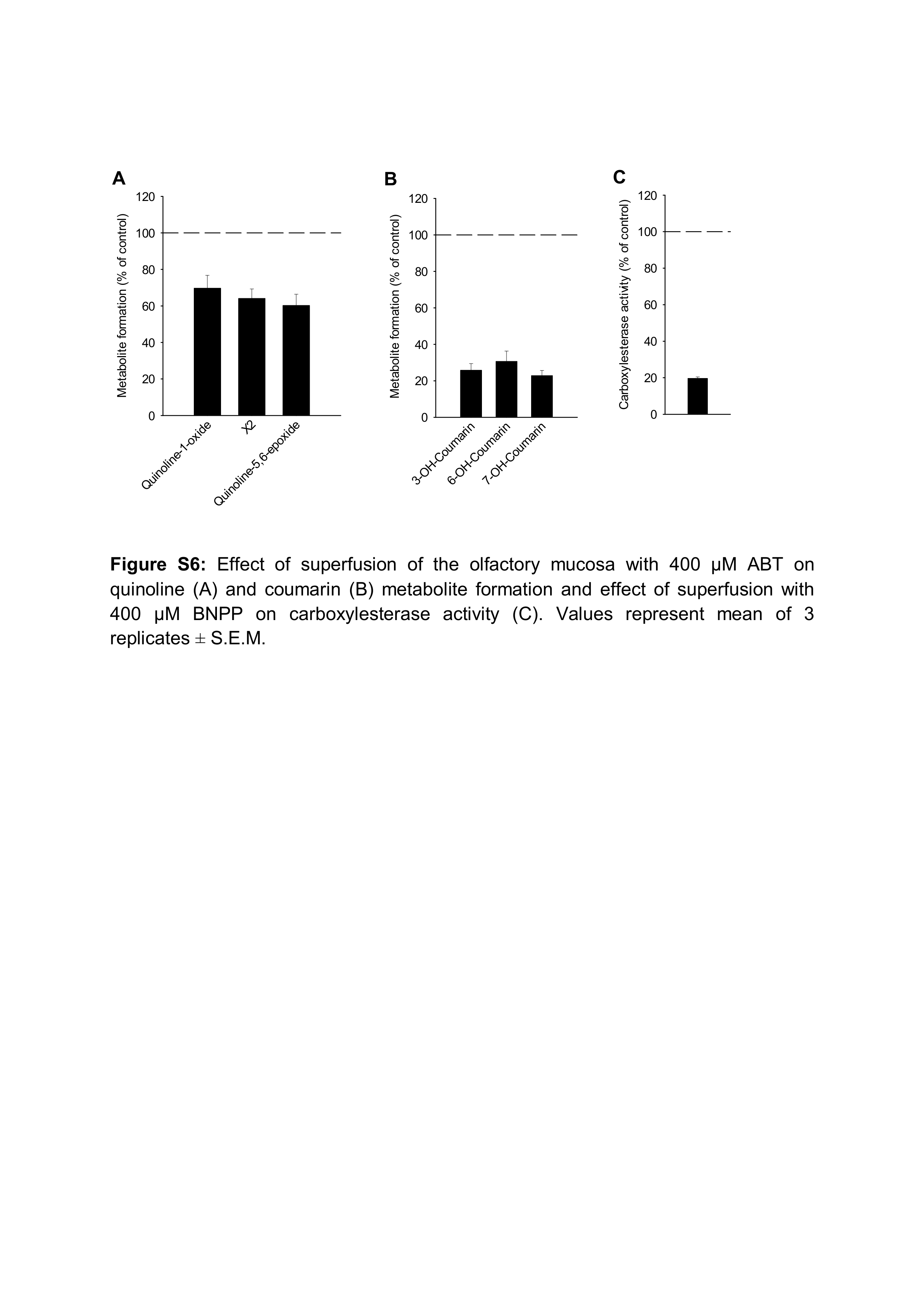

Supplement: Figure S6 — Effect of superfusion of the olfactory mucosa with 400 µM ABT on quinoline (A) and coumarin (B) metabolite formation and effect of superfusion with 400 µM BNPP on carboxylesterase activity (C). Values represent mean of 3 replicates ± S.E.M. (TIF) [file pone.0059547.s006.tif]

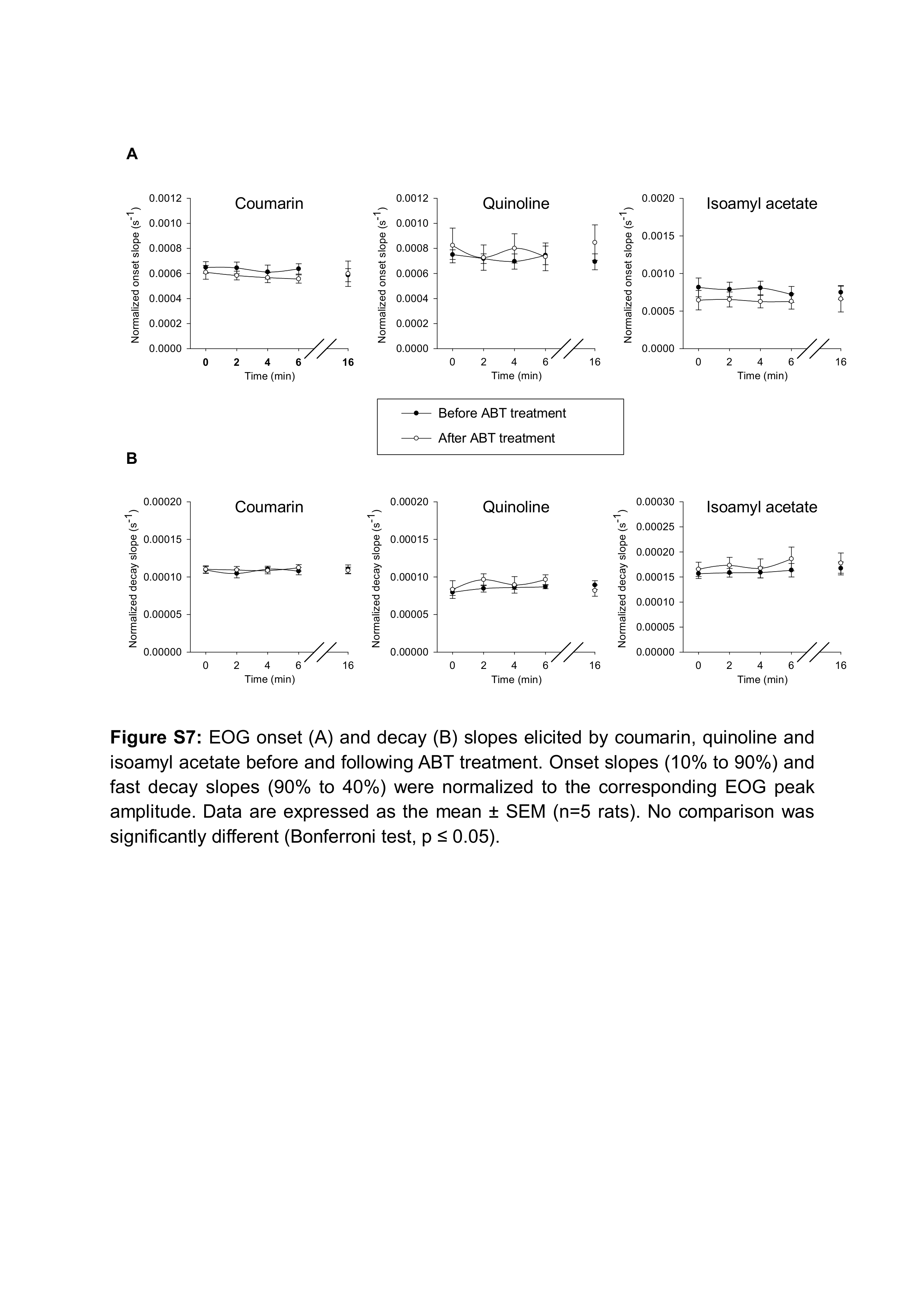

Supplement: Figure S7 — EOG onset (A) and decay (B) slopes elicited by coumarin, quinoline and isoamyl acetate before and following ABT treatment. Onset slopes (10% to 90%) and fast decay slopes (90% to 40%) were normalized to the corresponding EOG peak amplitude. Data are expressed as the mean ± SEM (n = 5 rats). No comparison was significantly different (Bonferroni test, p≤0.05). (TIF) [file pone.0059547.s007.tif]

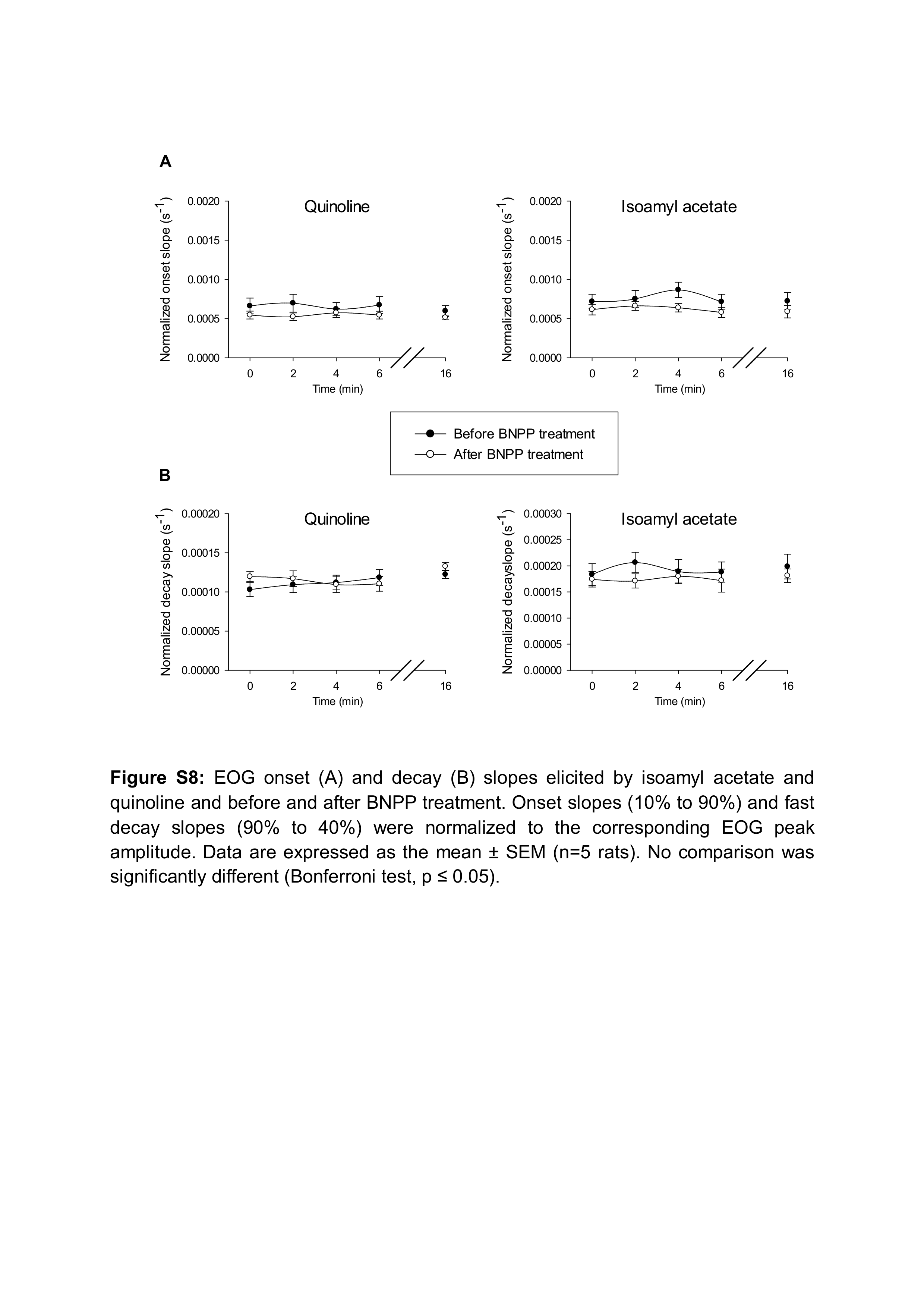

Supplement: Figure S8 — EOG onset (A) and decay (B) slopes elicited by isoamyl acetate and quinoline and before and after BNPP treatment. Onset slopes (10% to 90%) and fast decay slopes (90% to 40%) were normalized to the corresponding EOG peak amplitude. Data are expressed as the mean ± SEM (n = 5 rats). No comparison was significantly different (Bonferroni test, p≤0.05). (TIF) [file pone.0059547.s008.tif]
