# Supplementary material for: Odorant Metabolism Catalyzed by Olfactory Mucosal Enzymes Influences Peripheral Olfactory Responses in Rats
Source: PLoS One. 2013 Mar 26;8(3):e59547. doi: 10.1371/journal.pone.0059547 (PMC3608737; doi:10.1371/journal.pone.0059547)
Supplement: Table S1 — Characteristics of molecules used in the study. (PDF) [file pone.0059547.s009.pdf]

Table S1: Characteristics of molecules used in the study.

**Molecule** *(synonym)*	**CAS number**	**Molecular weight**	**Provider**
Quinoline	91-22-5	129.17	Sigma-Aldrich
3-Hydroxyquinoline	580-18-7	145.16	Interchim
8-Hydroxyquinoline	148-24-3	145.16	Sigma-Aldrich
8-Hydroxyquinoline-glucuronide	14683-61-5	321.3	Sigma-Aldrich
Quinoline-1-oxide (*Quinoline-N-oxide*)	64201-64-5	163.17	Interchim
Coumarin	91-64-5	146.15	Sigma-Aldrich
3-Hydroxycoumarin	939-19-5	162.16	Sigma-Aldrich
4-Hydroxycoumarin	1076-38-6	162.16	Sigma-Aldrich
6-Hydroxycoumarin	6093-68-1	162.16	Sigma-Aldrich
7-Hydroxycoumarin (*Umbelliferone*)	93-35-6	162.16	Sigma-Aldrich
4-Methylcoumarin	607-71-6	160.17	Sigma-Aldrich
4-Methylumbelliferone (*7-Hydroxy-4-methylcoumarin*)	90-33-5	176.17	Sigma-Aldrich
4-Methylumbelliferone-glucuronide (*7-Hydroxy-4-methylcoumarin-glucuronide*)	6160-80-1	352.3	Sigma-Aldrich
Isoamyl acetate	123-92-2	130.19	Sigma-Aldrich
Isoamylic alcohol	123-51-3	88.15	Merck
1-Aminobenzotriazole	1614-12-6	78.13	Sigma-Aldrich
Bis-*p*-nitro-phenylphosphate	645-15-8	340.18	Sigma-Aldrich
3-Isobutyl-1-methylxanthine	28822-58-4	222.3	Sigma-Aldrich
