# Supplementary material for: Odorant Metabolism Catalyzed by Olfactory Mucosal Enzymes Influences Peripheral Olfactory Responses in Rats
Source: PLoS One. 2013 Mar 26;8(3):e59547. doi: 10.1371/journal.pone.0059547 (PMC3608737; doi:10.1371/journal.pone.0059547)
Supplement: Table S2 — Incubation conditions used to study the in vitro metabolism of odorants. (PDF) [file pone.0059547.s010.pdf]

Table S2: Incubation conditions used to study the *in vitro* metabolism of odorants.

	**Coumarin**		**Quinoline**	**Isoamyl acetate**
**Subcellular fraction**	olfactory microsomes	hepatic microsomes	olfactory/hepatic microsomes	olfactory/hepatic S9
**Protein (mg/ml)**	0.3	1	1	0.5
**NADPH (mM)**	1	1	2	-
**$MgCl_2$ (mM)**	3	3	8	-
**Odorant (µM)**	250	50	250	5000
**Potassium phosphate buffer pH 7.4 (mM)**	100	100	100	100
**Final volume (µl)**	250	250	500	1000
