# Supplementary material for: Odorant Metabolism Catalyzed by Olfactory Mucosal Enzymes Influences Peripheral Olfactory Responses in Rats
Source: PLoS One. 2013 Mar 26;8(3):e59547. doi: 10.1371/journal.pone.0059547 (PMC3608737; doi:10.1371/journal.pone.0059547)
Supplement: Text S1 — Liquid chromatography - mass spectrometry analyses (PDF) [file pone.0059547.s011.pdf]

Text S1 : Liquid chromatography - mass spectrometry analyses

Instrumentation

Liquid chromatography (LC) was performed using a Dionex UltiMate™ 3000 LC pump from Thermo (San Jose, CA, USA) equipped with an autosampler and a diode array detector (DAD-3000). The separation was performed using a Hypersil Gold C18 Column (150 mm × 2.1 mm, i.d. × 3 µm) from Thermo (San Jose, CA, USA). The column temperature was maintained at 30°C. Injection volume was 10 µL. The mobile phase used to separate quinoline and its metabolites consisted of methanol (A) and water (B). The composition of solvent A varied as follows: 0-3 min, held at 5%; 12 min, 40%; 12-23 min, held at 40%; 24 min, 5%. The flow rate was 0.3 mL/min. The mobile phase used to separate coumarin and its metabolites consisted of acetonitrile (A) and water containing 0.01% formic acid (B). The composition of solvent A varied as follows: 0-7 min, held at 23%; 15 min, 45%; 18 min, 80%; 18-20 min, held at 80%; then immediate reduction to 23%. The flow rate was 0.2 mL/min.

Mass spectrometry (MS) was performed using a Thermo TSQ Quantum triple quadrupole mass spectrometer (San Jose, CA, USA) equipped with a standard electrospray ionisation source (ESI) outfitted with a 100-µm i.d. H-ESI needle. The source spray head was oriented at a 90° angle orthogonal to the ion-transfer tube. Nitrogen was used for both the sheath and the auxiliary gases. The MS signals of quinoline and coumarin metabolites were first optimised by continuous infusion of the standards dissolved in the mobile phase using ESI in negative and positive modes.

For analysis of quinoline metabolites, the electrospray ionisation spray voltages were 4 kV in positive ion mode, vaporiser temperature was 200°C, sheath gas N2 pressure 20 (arbitrary units), auxiliary gas pressure 0 (arbitrary units), ion sweep gas pressure 5, ion transfer capillary temperature 300°C, skimmer offset 10 V and multiplier gain 300,000. For analysis of coumarin metabolites, the electrospray ionisation spray voltages were 3.5 kV in negative ion mode, vaporiser temperature was 100°C, sheath gas N2 pressure 20 (arbitrary units), auxiliary gas pressure 10 (arbitrary units), ion sweep gas pressure 5, ion transfer capillary temperature 300°C, skimmer offset 15 V and multiplier gain 300,000.

When operated under full scan product conditions in the negative and positive ion modes, data were collected in the range from 50 to 250 Da with a scan time of 0.5 s. For characterization of quinoline and its metabolites in the positive mode, ESI-MS/MS was used with argon as the collision gas at 1.5 mTor, and the collision energy was set to 33 eV for MS^2 of m/z 130, to 25 eV for MS^2 of m/z 146 and to 32 eV MS^2 of m/z 162. For coumarin metabolite characterization in the negative mode, ESI-MS/MS was used with argon as the collision gas at 1.5 mTor, and the collision energy was set to 15 eV for MS^2 of m/z 161. The data were processed using the Xcalibur software (Thermo).

Results

Incubations were performed as described in the section Material and methods.

Analysis of quinoline metabolites.

LC-ESI-MS/MS analysis of a mixture containing quinoline and quinoline-1-oxide by scan product mode of the molecular ion under the positive ionization mode gave $[M+H]^+$ ions at m/z 130 and 146, respectively (supplementary Fig. S1A).

The UV-chromatogram obtained from incubation of quinoline with olfactory microsomes was close to that obtained previously (cf. Fig. 1A). Four main metabolites were detected (Qm1,

Qm2, Qm3 and Qm4) (supplementary Fig. S1B). The positive ESI-MS analysis of these metabolites gave $[M+H]^+$ ions at m/z 146, indicating that these compounds are oxygenated metabolites. The MS^2 spectrum of the selected molecular ion $[M+H]^+$ of Qm2 (RT = 10.8 min) at m/z 146 corresponded to that of quinoline-1-oxide (supplementary Fig. S2). In the absence of authentic standards, formal identification of other metabolites was not possible. However, comparison of the UV spectrum of Qm3 (RT = 11.4 min) with spectra of quinoline derivatives published by Saeki et al. [21] led us to assume that Qm3 would be quinoline-5-6-epoxide.

Metabolites with $[M+H]^+$ ions at m/z 162, which eluted between 6 and 9.5 min, were also detected by mass spectrometry (supplementary Fig S1B and Fig. S3). These compounds might be diols (M+1+32).

Analysis of coumarin metabolites

LC-ESI-MS/MS analysis of a mixture containing authentic standards (coumarin, 3-hydroxycoumarin, 6-hydroxycoumarin and 7-hydroxycoumarin) by scan product mode of the molecular ion $[M-H]^-$ at m/z 161 under the negative ionization mode showed that the hydroxylated derivatives 6-hydroxycoumarin and 7-hydroxycoumarin are well detected whereas 3-hydroxycoumarin was slightly detected at m/z 161 and coumarin was not detected in this condition (supplementary Fig S4A). The single MS spectrum of 3-hydroxycoumarin showed a major fragment at m/z 133 indicating that this compound fragmented in the source during ionization (supplementary Fig S5A).

The UV-chromatogram obtained from incubation of coumarin with olfactory microsomes was quite similar to that obtained in the first experiment (cf. Fig. 3A). Four major metabolites were detected (Cm1, Cm2, Cm3 and Cm4) (supplementary Fig S4B). The negative single MS analysis of these metabolites gave $[M-H]^-$ ions at *m/z* 161, indicating that these compounds corresponded to different isomers of hydroxylated coumarin. Moreover, based on our data obtained by MS^2 analysis of authentic standards, the metabolites Cm1, Cm2 and Cm4 were identified as 7-hydroxycoumarin, 6-hydroxycoumarin and 3-hydroxycoumarin, respectively (supplementary Fig. S5). The MS^2spectrum of the selected molecular ion $[M+H]^+$ of Cm3 at m/z 161 exhibited a main fragment ion at m/z 133. Cm3 might be 5- or 8-hydroxycoumarin but these compounds are not commercially available to verify this hypothesis.
